# Ramified derivatives of 5-(perylen-3-ylethynyl)uracil-1-acetic acid and their antiviral properties†

**DOI:** 10.1039/c9ra06313g

**Published:** 2019-08-20

**Authors:** Ksenia A. Sapozhnikova, Nikita A. Slesarchuk, Alexey A. Orlov, Evgeny V. Khvatov, Eugene V. Radchenko, Alexey A. Chistov, Alexey V. Ustinov, Vladimir A. Palyulin, Liubov I. Kozlovskaya, Dmitry I. Osolodkin, Vladimir A. Korshun, Vladimir A. Brylev

**Affiliations:** Shemyakin-Ovchinnikov Institute of Bioorganic Chemistry Miklukho-Maklaya 16/10 Moscow 117997 Russia v-korshun@yandex.ru; Department of Chemistry, Lomonosov Moscow State University Moscow 119991 Russia; FSBSI "Chumakov FSC R&D IBP RAS" Poselenie Moskovsky Moscow 108819 Russia; Sechenov First Moscow State Medical University Moscow 119991 Russia dmitry_o@qsar.chem.msu.ru; Biotech Innovations Ltd Leninskie Gory 1 bd 75 Moscow 119992 Russia; Department of Biology and Biotechnology, National Research University Higher School of Economics Vavilova 7 Moscow 117312 Russia

## Abstract

The propargylamide of N3-Pom-protected 5-(perylen-3-ylethynyl)uracil acetic acid, a universal precursor, was used in a CuAAC click reaction for the synthesis of several derivatives, including three ramified molecules with high activities against tick-borne encephalitis virus (TBEV). Pentaerythritol-based polyazides were used for the assembly of molecules containing 2⋯4 antiviral 5-(perylen-3-ylethynyl)uracil scaffolds, the first examples of polyvalent perylene antivirals. Cluster compounds showed enhanced absorbance, however, their fluorescence was reduced due to self-quenching. Due to the solubility issues, Pom group removal succeeded only for compounds with one peryleneethynyluracil unit. Four compounds, including one ramified cluster 9f, showed remarkable 1⋯3 nM EC_50_ values against TBEV in cell culture.

## Abstract 2

Ramified clusters of antiviral perylenylethynyl scaffold were prepared using CuAAC reaction of 5-(perylen-3-ylethynyl)-3-pivaloyloxymethyl-1-(propargylamidomethyl)uracil with azides. Compounds inhibited TBEV reproduction at nanomolar concentrations.

## Graphical abstract

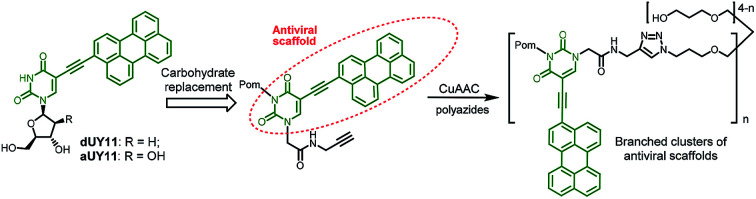

## Introduction

**Fig. 1:**
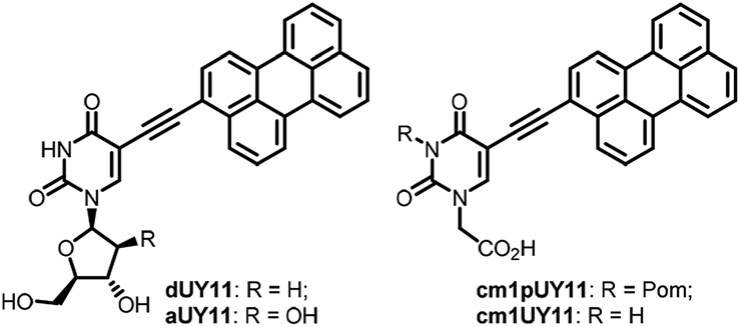
Structures of broad spectrum antiviral peryleneethynyluracil compounds.

5-(Perylen-3-ylethynyl)-*deoxy*-uridine dUY11 (Fig. 1), prepared initially as a fluorescent nucleoside,^1^ had unexpectedly shown remarkably broad spectrum antiviral properties.^2^ Nucleoside derivative dUY11 and its arabino analog aUY11 effectively suppressed reproduction of a number of enveloped viruses in cell cultures, thus constituting a new class of antivirals with a non-nucleoside mechanism of action.^2,3^ The presumable target for these compounds is the lipid membrane bilayer of the virion envelope. Compounds dUY11 and aUY11 are rigid amphipathic fusion inhibitors (RAFIs)^2*c*^ that target the viral envelope either by a shape-determined biophysical mechanism^2*c*,3,4^ or by photosensitization.^5^ Both shape-determined and photophysical properties of the conjugated aryl part of the molecule are probably crucial for the antiviral properties, and the carbohydrate part seems to be less important. Very recently,^6^ we prepared 5-(perylen-3-ylethynyl)uracil-1-acetic acid (cm1UY11, Fig. 1) with a carboxymethyl group replacing the pentose moiety. The acid showed a pronounced antiviral activity against tick-borne encephalitis virus (TBEV),^6^ herpes simplex virus type 1 (HSV-1),^7^ and African swine fewer virus (ASFV).^8^ Moreover, its 3-Pom-modified precursor cm1pUY11 (Fig. 1) and amides were also active against TBEV^6^ and HSV-1.^7^

Thus, the 5-(perylen-3-ylethynyl)uracil scaffold seems to be responsible for the antiviral activity of perylene RAFIs. Lipophilic perylene residue is expected to reside in the lipid bilayer upon binding between a RAFI molecule and an enveloped virion. Therefore, one can expect cooperative enhancement of lipid membrane anchoring for compounds containing 2⋯4 perylene residues, or other effects of clustering perylenes.

The concept of clusterized/multivalent (or dendrimeric) antivirals has been developed for decades,^9^ both for specific and non-specific compounds and functional groups. Recent promising examples include polyanions,^10^ polycations,^11^ as well as dendrimers carrying amino acids,^12^ peptides,^13^ phenols,^14^ terpenes,^15^ mono-,^16^ oligosaccharides,^17^ and neuraminidase inhibitors.^18^ The approach, however, has been never applied to RAFIs.

To study the possible effects of combining several perylene cores in a single molecule on the antiviral activity, we synthesized small clusters of 5-(perylen-3-ylethynyl)-1-(carboxymethyl)uracil, quantified their fluorescence properties, and measured the efficiency of TBEV reproduction inhibition by these clusters in PEK cell culture.

## Results and discussion

### Synthesis of compounds

**Scheme 1:**
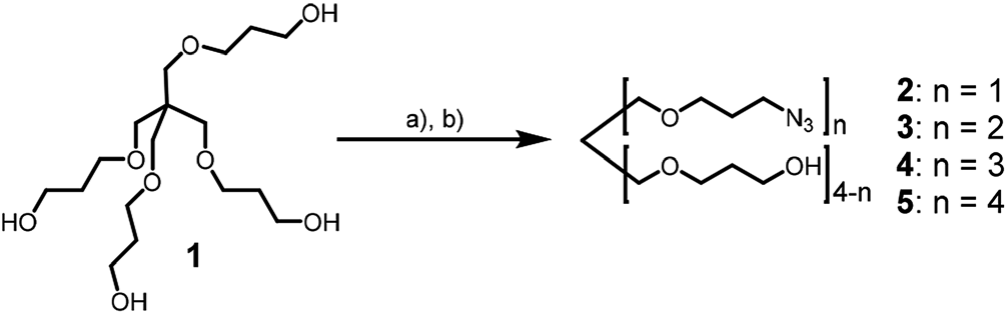
Synthesis of azides. Conditions: (a) methanesulfonyl chloride, NEt_3_, DCM; (b) NaN_3_, DMSO, rt, 12 h, 32% (2), 27% (3), 8% (4).

Recently, we used the azide–alkyne click reaction for the synthesis of dUY11 derivatives with enhanced activity.^19^ Here we report the application of Huisgen–Meldal–Sharpless reaction (Cu(i)-catalyzed alkyne–azide cycloaddition, CuAAC)^20^ for the synthesis of 5-(perylen-3-ylethynyl)-1-(carboxymethyl)uracil derivatives. First, we prepared a set of branched azides 2–5 (Scheme 1). The starting tetraol 1 was mesylated with a controlled excess of mesyl chloride (MsCl) in DCM in the presence of triethylamine as a base. Tuning the mesyl chloride/hydroxyl ratio leads to the desired distribution of products in the reaction. Earlier, we prepared azides 4 and 5 using 1 : 3 molar ratio of 1 to mesyl chloride^21^ and used them for the assembly of complex oligonucleotide conjugates.^21,22^ Here we report the synthesis of azides 2 and 3 as main products. Mesylation with 1 : 1.2 molar ratio (1 : MsCl), and subsequent nucleophilic substitution with sodium azide affords compounds 2 and 3 in 32% and 27% yield, respectively, together with some amount of triazide 4.

**Scheme 2:**
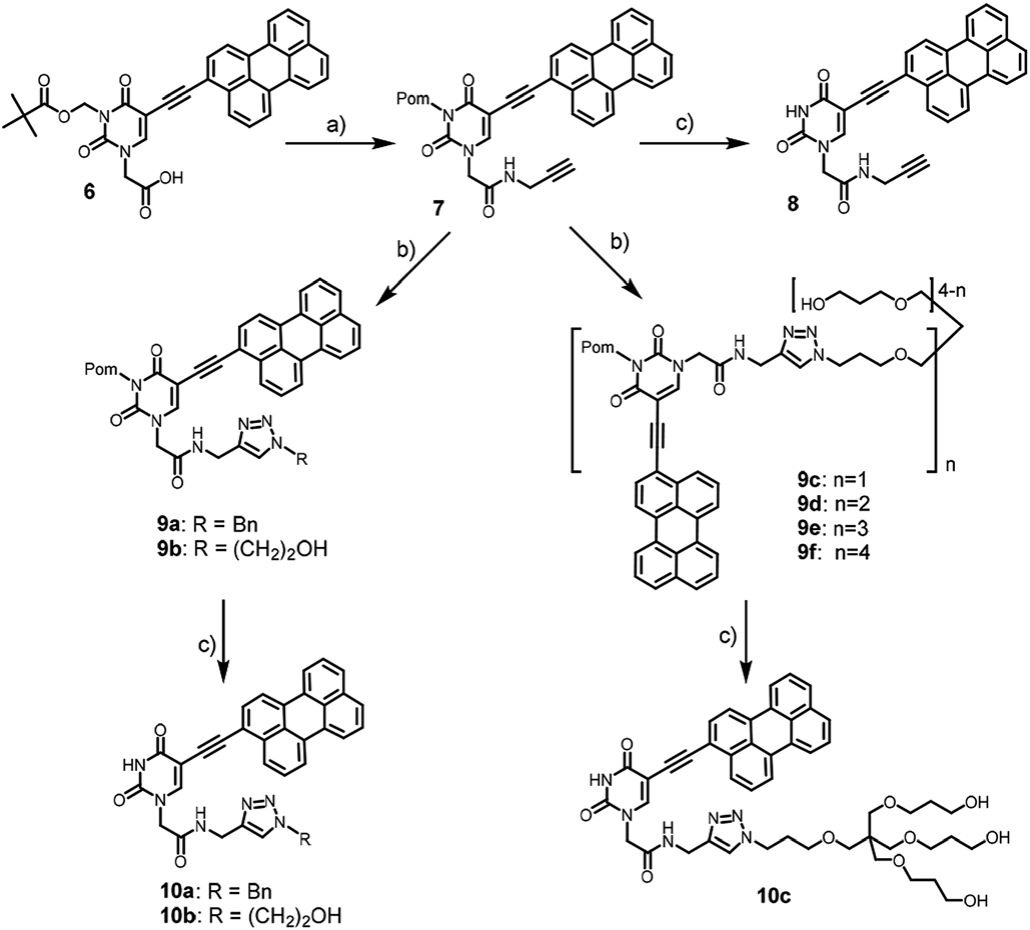
Synthesis of the model and target compounds. Conditions: (a) propargylamine, PyBOP, DIPEA, DMF, 0 °C, 55%; (b) CuSO_4_·5H_2_O, TBTA, aq. ascorbic acid, DMSO, rt, 12 h, azide (benzylazide, azidoethanol, or 2–5), 75% (9a); 67% (9b); 81% (9c), 70% (9d), 54% (9e), 38% (9f); (c) NaOH, DMSO, MeOH, rt, 30 min, 35% (10a), 40% (10b), 30% (10c).

We used pivaloyloxymethyl (Pom) protection for N3 position of the nucleobase for solubility reasons. 3-Pom-5-(perylen-3-ylethynyl)uracil acetic acid 6 was prepared from 5-iodouracil in several steps.^6^ It was coupled with propargylamine to yield alkyne precursor 7, which was conjugated with benzyl azide, azidoethanol, and pentaerythritol-based azides 2–5 (Scheme 2) *via* CuAAC to afford compounds 9.

The alkaline removal of pivaloyloxymethyl (Pom) protection from compounds 7 and 9a–c yielded the corresponding amides 8, 10a–c (Scheme 2). However, the usual Pom group deprotection protocol with sodium methoxide or ammonia in methanol^23^ was inappropriate because both the starting compounds and the desired products were insoluble in alcohol. Therefore, we modified the deprotection protocol by choosing NaOH as the base, and DMSO/MeOH/H_2_O mixture as the reaction medium. As a result, full conversion of the starting material occurred in 30 min (TLC control).

While the conversions of compounds 9a–c into 10a–c by TLC were excellent, their isolated yields in deprotection steps (Scheme 2) turned out to be poor because of the losses during the purification using column chromatography. The products showed low solubility and chromatographic isolations were carried out in the overloading mode. Thus, a number of fractions containing the products were overlapping with some minor impurities, and they were excluded to obtain high quality samples for antiviral activity studies.

We failed to prepare and isolate Pom-deprotected compounds from 9d–f because of the incomplete deprotection, and low solubility of the desired products.

### Spectral studies

**Fig. 2:**
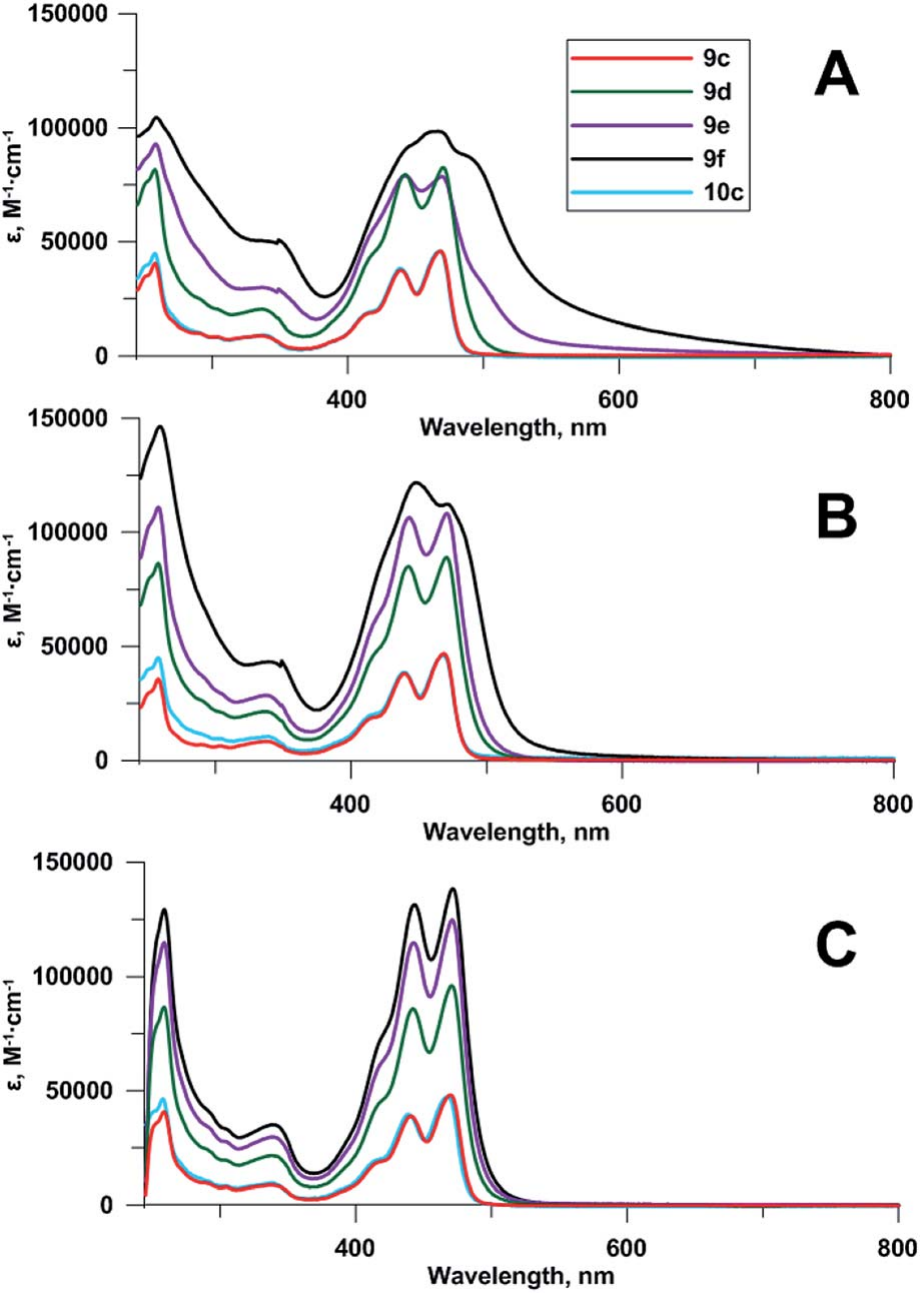
Absorbance spectra of compounds 9c–f, 10c at 5 μM concentrations: (A) 0.2% DMSO in 96% EtOH, (B) 2% DMSO in 96% EtOH, (C) 20% DMSO in 96% EtOH.

Compounds 9d–f contain 2⋯4 chromophoric units linked with flexible spacers. To obtain some insight into the structure of the compounds in a solution and to reveal a possible influence of spectral properties on antiviral activity,^2*d*^ we compared their spectra to each other, and to those of monomeric compounds 9c and 10c. UV-Vis spectra (Fig. 2) showed no significant difference between Pom-protected compound 9c and the deprotected derivative 10c; the shape of the spectrum, and the positions of maxima were always identical.

The increase of the number of chromophoric units led to the corresponding increase of the molar attenuation coefficient in true solution (20% DMSO in 96% ethanol as a solvent). Upon the reduction of DMSO concentration to 2% and further to 0.2%, the bands in absorbance spectra of tetramer 9f, and then of trimer 9e became broader and less structured (Fig. 2). The effect, confirming the aggregation of chromophoric residues, was observed earlier for other perylene compounds.^19,24^

**Fig. 3:**
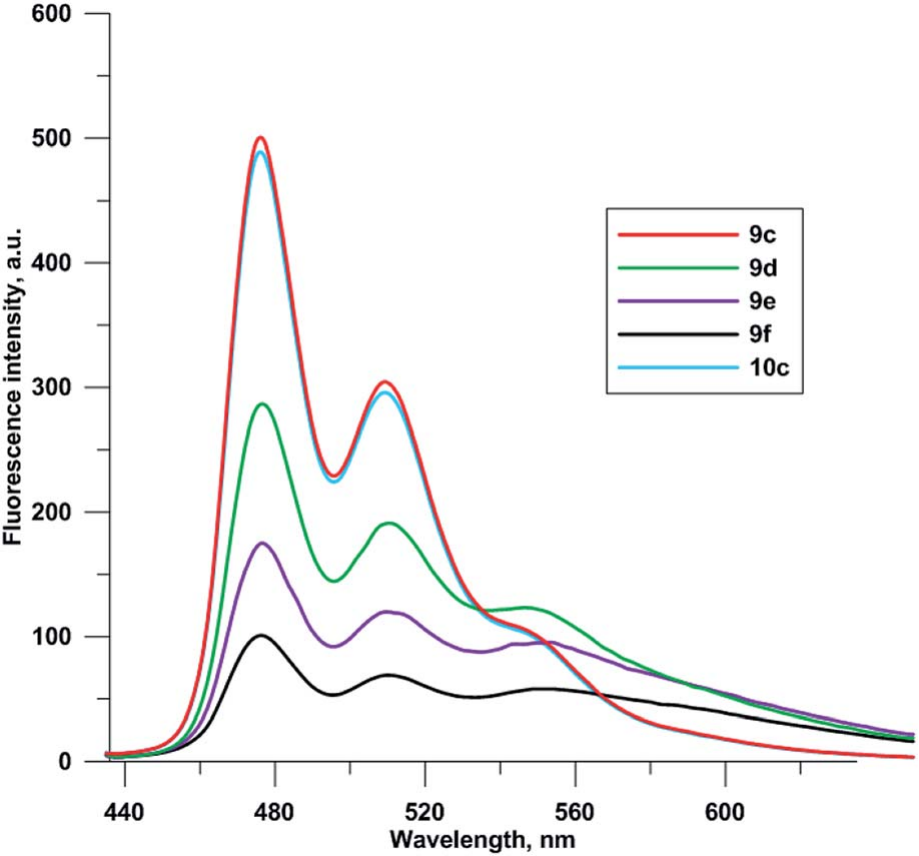
Fluorescence spectra of 9c–f, 10c (0.1 μM, 96% EtOH, *λ*_ex_ 420 nm).

Fluorescence spectra of compounds 9c–f and 10c were recorded at 0.1 μM concentrations in 96% EtOH (Fig. 3). Due to self-quenching, fluorescence intensity decreases with the increase in the number of fluorophores comprising the 5-(perylen-3-ylethynyl)uracil groups. There were no drastic changes in fluorescence spectra for compounds 9c–f and 10c, except for relative enhancement of 550 nm band, and proximal long wavelength emission for polychromophore molecules 9d–f, suggesting weak excimer fluorescence from perylene residues located close to each other.

### Antiviral properties

All prepared compounds, containing one (7–9b, 10a–c) or several (9d–f) perylene residues, were tested for cytotoxicity and for ability to inhibit TBEV (strain Absettarov) reproduction in PEK cell culture as measured by plaque reduction assay (Table 1). All experimental conditions were the same as in our previous studies.^2*d*,6,19,25^

**Table 1:** TBEV (strain Absettarov) reproduction inhibition efficiency (EC_50_) and cytotoxicity (CC_50_, PEK cells) of the compounds 6–10

#	EC_50_, μM	CC_50_, μM	
		24 h	7 d
6	0.00121 ± 0.00025^a^	>50	>50
7	0.00097 ± 0.00015	>50	>50
8	0.002 ± 0.001	>50	<50
9a	0.046 ± 0.017	>50	>50
9b	0.0010 ± 0.0003	>50	>50
9c	0.008 ± 0.001	>50^b^	18
9d	0.024 ± 0.007	>50^b^	<50
9e	0.015 ± 0.001	>50^b^	<50
9f	0.0033 ± 0.0011	>50	>50
10a	0.0081 ± 0.0017	>50^b^	>50^b^
10b	0.066 ± 0.013	>50	>50
10c	0.10 ± 0.05	>50	37 ± 13

a Data for compound 6 are from ref. 6.

b Morphological changes of cell membranes were observed.

All the compounds showed little to no cytotoxicity on PEK cells and selectivity indices (SI = CC_50_/EC_50_) up to 50 000. The alkyne precursor 7 appeared to be the most potent compound in the series, showing nanomolar EC_50_. Its unprotected analogue 8 and acid precursor 6 showed slightly lower activity, thus revealing low influence of small substituents in the uracil moiety, similarly to one of the previous studies,^25*a*^ but contrary to another.^6^ Although cluster compounds 9d–f inhibited TBEV reproduction in one or two digit nanomolar concentrations, their single-perylene analogues were more potent. Remarkably, Pom deprotection of branched compound 9c, affording 10c, led to a considerable activity decrease.

### Molecular modeling

**Fig. 4:**
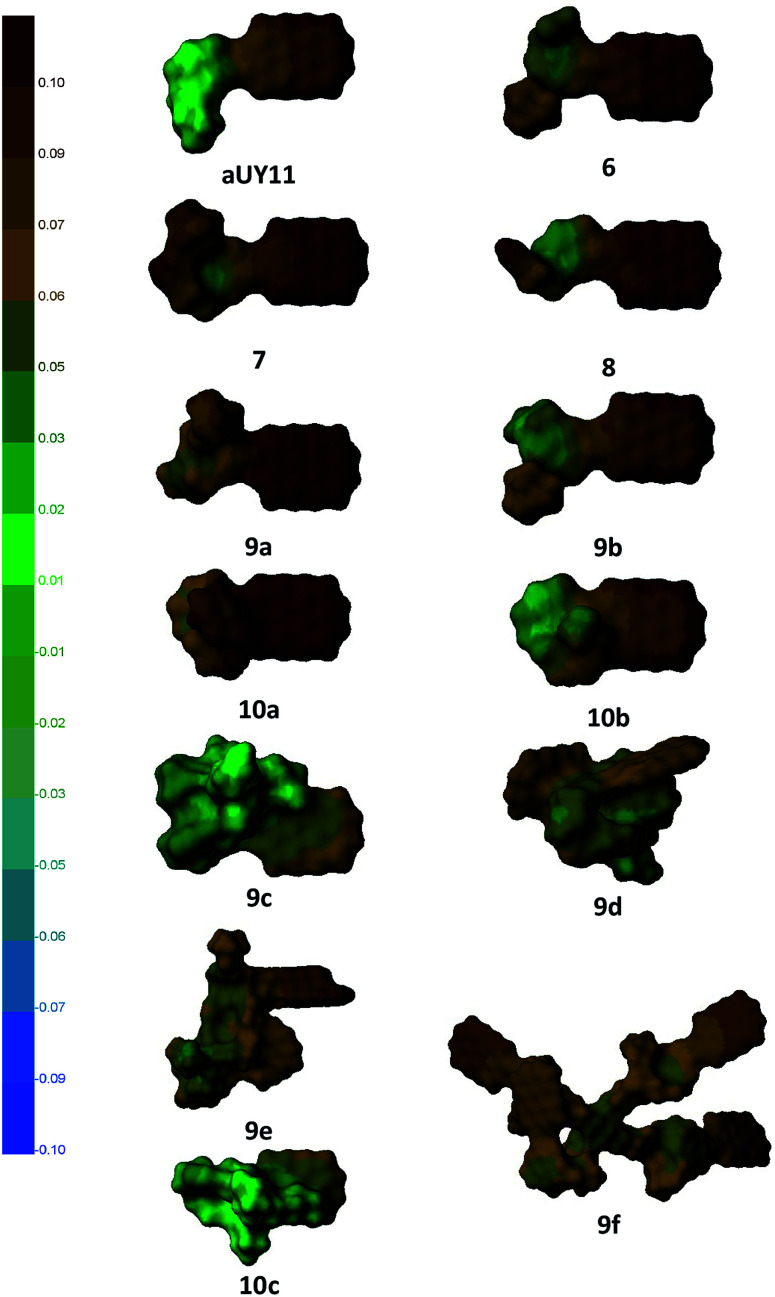
Structures of compounds optimized using SYBYL-X 2.1 software; molecular surfaces are colored by the hydrophobic potential (left scale).

While the abbreviation RAFI stands for *Rigid* Amphipathic Fusion Inhibitor, the compounds synthesized here contain a large number of rotatable bonds. Thus, their antiviral activity and membrane interactions may be largely defined by their conformational space. To obtain some insight into this conformational space, we performed a simple conformational analysis for molecular models of the compounds, seeking the global minima of the compound energies using the Confort method^26^ implemented in SYBYL-X 2.1.^27^ The optimized structures are shown in Fig. 4, along with the molecular surfaces colored by the hydrophobic potential.

As the most important part of the RAFI molecules is the perylene moiety,^2*d*,25*a*^ its exposure and ability to interact with the viral membrane should play a crucial role in the potency of the compounds. It can be seen that compounds 6–8, as well as aUY11, do not show substantial flexibility, and the perylene moiety of these compounds is completely exposed. Molecules 9 and 10, bearing longer and more flexible moieties, show less uniform antiviral activity, which is more strongly affected by possible conformational behavior. For example, the influence of Pom is opposite in the pairs 9a–10a and 9b–10b; in the former, the benzyl moiety may interact with perylene, whereas in the latter the hydroxyethyl group should prefer a more hydrophilic environment.

Using coloring by the hydrophobic potential, one can see the amphipathic nature of perylenylethynyluracil compounds. Perylenylethynyluracil units are probably responsible for lipid bilayer anchoring and for the general antiviral effect. The polar part of the molecules seems to have a modulating (within two orders of magnitude)^6^ effect on the inhibition of a viral replication. Remarkably, in the most active compounds, the polar part of the molecule has a small hydrophobic unit, Pom or benzyl, but not both together. Although neither the target, nor the mechanism of antiviral action of RAFIs was directly proven to date, this simple observation can be used in a future design of antivirals.

## Experimental

### General methods

Reagents and solvents were from commercial suppliers and used as received, except DMF, DMSO, CH_2_Cl_2_, CHCl_3_, EtOH and methanol were used freshly distilled from CaH_2_. Azides 4, 5,^21^ tetrakis(5-hydroxy-2-oxapentyl)methane^21^ and 3-Pom-5-(perylen-3-ylethynyl)uracil-1-acetic acid 6^6^ were prepared as described. 500 MHz ^1^H and 125.7 MHz ^13^C NMR spectra were recorded on Bruker AMX-400 or Bruker Avance 500 spectrometers and referenced to DMSO-*d*_6_ (2.50 ppm for ^1^H and 39.5 ppm for ^13^C) or CDCl_3_ (7.26 ppm for ^1^H and 77.16 ppm for ^13^C). ^1^H NMR coupling constants are reported in hertz (Hz) and refer to apparent multiplicities. Electrospray ionization high resolution mass spectra (ESI HRMS) of low molecular weight compounds were recorded using a Thermo Scientific Orbitrap Exactive mass spectrometer (positive or negative ion mode). UV spectra were recorded on a Varian Cary 100 spectrophotometer. Fluorescence spectra were recorded using a PerkinElmer LS 55 fluorescence spectrometer. Analytical thin layer chromatography was performed on Kieselgel 60 F_254_ precoated aluminum plates (Merck). Silica gel column chromatography was performed using Merck Kieselgel 60 0.040–0.063 mm.

### Cell culture and viruses

Porcine embryo kidney (PEK) cell line was maintained at 37 °C in medium 199 (FSBSI "Chumakov FSC R&D IBP RAS", Russia) supplemented with 5% fetal bovine serum (Gibco). Tick-borne encephalitis virus strain Absettarov (GenBank access no. KU885457) was from the laboratory collection of FSBSI "Chumakov FSC R&D IBP RAS".

### Synthetic procedures

#### 6,6-Bis(5-hydroxy-2-oxapentyl)-4,8-dioxa-11-hydroxyundec-1-yl azide (2)

Mesyl chloride (7.06 g, 60 mmol) was added dropwise to a mixture of tetrakis(5-hydroxy-2-oxapentyl)methane (17.26 g, 50 mmol) and triethylamine (11.1 mL, 80 mmol) in dry DCM (300 mL). The reaction was monitored by TLC (10% EtOH in CHCl_3_; starting alcohol: *R*_f_ 0.33). After the consumption of all starting material, the reaction mixture was washed with distilled water (2 × 100 mL), brine (3 × 50 mL) and dried over Na_2_SO_4_. Evaporation of the solvent in vacuum gave yellowish oily liquid. It was dissolved in dry DMSO (60 mL), and sodium azide (10 g; 154 mmol) was added under magnetic stirring. After 12 h, water (120 mL) was added and the mixture of products was extracted from the aqueous layer with EtOAc (4 × 100 mL); organic fractions were combined, washed with distilled water (5 × 100 mL), brine (3 × 100 mL) and dried over Na_2_SO_4_. After evaporation of the solvent in vacuum, the residue was chromatographed on silica gel (gradient elution with 20 → 45% of 96% ethanol in CHCl_3_). Compound 2c (5.24 g, 32% yield) was obtained as a colorless viscous liquid. *R*_f_ 0.34 (5% EtOH in CHCl_3_). ^1^H NMR (500 MHz; CDCl_3_) *δ* 3.71 (t, 6H, *J* = 5.5 Hz), 3.55 (t, 6H, *J* = 5.56 Hz), 3.47 (br s, 3H), 3.44 (t, 2H, *J* = 5.97 Hz), 3.38 (s, 6H), 3.36–3.32 (m, 4H), 1.84–1.74 (m, 8H). ^13^C NMR (125 MHz; CDCl_3_) *δ* 70.9, 70.5, 70.4, 68.1, 61.5, 48.5, 44.8, 31.8, 29.0. HRMS (ESI, *m*/*z*): calcd for C_17_H_36_N_3_O_7_ ([M + H]^+^): 394.2548, found: 394.2557. Earlier eluting diazide 3 (4.7 g; 27%) and triazide 4 (1.43 g; 8%) were obtained as colorless viscous liquids. Their analytical data matched with ones reported earlier.^21^

#### [3-(Pivaloyloxymethyl)-5-(perylen-3-ylethynyl)uracil-1-yl]-*N*-propargylacetamide (7)

To an ice-cooled magnetically stirred solution of acid 6 (255 mg; 0.46 mmol) in dry DMF (10 mL) PyBOP (273 mg; 0.52 mmol) and DIPEA (158 μL; 0.9 mmol) were consequently added under argon atmosphere. After 1 min, propargylamine (38 μL; 0.6 mmol) was added, the reaction mixture stirred for next 10 min and quenched with water (20 mL). The product was extracted using EtOAc (4 × 30 mL). The organic layer was washed with water (3 × 10 mL), brine (2 × 20 mL) and dried over Na_2_SO_4_. Evaporation of the solvent gave orange-brownish solid which was purified by column chromatography on silica gel (EtOH 1 → 7% gradient in CH_2_Cl_2_). Compound 7 was isolated as orange solid (151 mg; 55% yield). *R*_f_ 0.34 (5% EtOH in CHCl_3_). UV-Vis (20% DMSO in 96% EtOH) *λ*_max_, nm (*ε*, M^−1^ cm^−1^): 441 (38 600), 470 (48 000), *λ*_min_, nm (*ε*, M^−1^ cm^−1^): 452 (26 800); fluorescence (96% EtOH, *λ*_em_ 530 nm, excitation *λ*_max_, nm): 334, 440, 467; (96% EtOH, *λ*_ex_ 420 nm, emission *λ*_max_, nm): 476, 509. ^1^H NMR (500 MHz; DMSO-*d*_6_) *δ* 8.78 (t, 1H, *J* = 5.35 Hz), 8.43 (s, 1H), 8.38 (d, 1H, *J* = 7.55 Hz), 8.36–8.27 (m, 3H), 8.25 (d, 1H, *J* = 8.23 Hz), 7.82–7.77 (m, 2H), 7.68–7.62 (m, 2H), 7.53 (t, 2H, *J* = 7.68 Hz), 5.86 (s, 2H), 4.53 (s, 2H), 3.99–3.94 (m, 2H), 3.2–3.18 (m, 1H), 1.14 (s, 9H). ^13^C NMR (125 MHz; DMSO-*d*_6_) *δ* 176.6, 166.0, 160.5, 149.7, 149.3, 134.1, 133.7, 131.3, 131.0, 130.5, 130.1, 129.8, 128.6, 128.3, 127.8, 127.7, 127.6, 127.0, 126.9, 125.6, 121.6, 121.4, 121.2, 120.2, 119.1, 96.8, 90.9, 87.8, 80.6, 73.5, 65.1, 50.8, 38.3, 28.1, 26.7. HRMS (ESI, *m*/*z*): calcd for C_37_H_29_N_3_O_5_Na ([M + Na]^+^): 618.1999, found: 618.2000.

### Click reaction between alkyne 7 and mono-azides, a general procedure

Azide (0.30 mmol) and propargyl amide 7 (0.26 mmol) were dissolved in DMSO (10 mL) under vigorous magnetic stirring. The solution was flushed with argon for degassing. 0.1 M solution of CuSO_4_·5H_2_O and TBTA ligand (1 : 1) in 55% DMSO was added (260 μL). Then ascorbic acid (100 mg mL^−1^ in H_2_O; 91 μL) was quickly loaded and the flask was tightly closed. After 12 h TLC showed full consumption of the starting alkyne 7. Reaction mixture was quenched with brine (50 mL) and lumpy solid precipitate was isolated by centrifugation. The crude product was washed with distilled water (2 × 10 mL). The residue was applied on the silica gel using evaporation of the acetone solution with small portion of the sorbent. The obtained powder was carefully spilled on the column filled with silica gel in CH_2_Cl_2_. The following compounds were isolated.

#### [3-(Pivaloyloxymethyl)-5-(perylen-3-ylethynyl)uracil-1-yl]-*N*-(1-benzyl-1,2,3-triazol-4-yl)acetamide (9a)

[3-(Pivaloyloxymethyl)-5-(perylen-3-ylethynyl)uracil-1-yl]-*N*-(1-benzyl-1,2,3-triazol-4-yl)acetamide (9a) was prepared using benzyl azide. Gradient elution with EtOH (1 → 5%) in CH_2_Cl_2_ and evaporation gave pure compound as orange solid, yield 137 mg (75%). *R*_f_ 0.32 (5% EtOH in CH_2_Cl_2_); UV-Vis (20% DMSO in 96% EtOH) *λ*_max_, nm (*ε*, M^−1^ cm^−1^): 441 (38 600), 470 (48 000), *λ*_min_, nm (*ε*, M^−1^ cm^−1^): 452 (26 800). Fluorescence (96% EtOH, *λ*_em_ 530 nm, excitation *λ*_max_, nm): 334, 440, 467; (96% EtOH, *λ*_ex_ 420 nm, emission *λ*_max_, nm): 476, 509. ^1^H NMR (500 MHz, DMSO-*d*_6_) *δ* 8.79–8.73 (m, 1H), 8.42–8.38 (m, 2H), 8.38–8.24 (m, 4H), 7.99 (s, 1H), 7.83–7.78 (m, 2H), 7.7–7.63 (m, 2H), 7.54 (t, 2H, *J* = 7.62 Hz), 7.39–7.28 (m, 6H), 5.88 (s, 2H), 5.58 (s, 2H), 4.54 (s, 2H), 3.3–3.27 (m, 2H), 1.14 (s, 9H). ^13^C NMR (125 MHz, DMSO-*d*_6_) *δ* 176.6, 166.1, 160.5, 149.7, 149.4, 144.5, 136.0, 134.2, 133.7, 131.3, 131.0, 130.5, 130.0, 129.8, 128.7, 128.7, 128.3, 128.1, 128.0, 127.9, 127.7, 127.6, 127.0, 126.9, 125.6, 123.1, 121.6, 121.4, 121.3, 120.3, 119.1, 96.8, 90.9, 87.9, 65.1, 52.8, 51.0, 38.3, 34.4, 26.7. HRMS (ESI, *m*/*z*): calcd for C_44_H_36_N_6_O_5_Na ([M + Na]^+^): 751.2639, found: 751.2629.

#### [3-(Pivaloyloxymethyl)-5-(perylen-3-ylethynyl)uracil-1-yl]-*N*-[1-(2-hydroxyethyl)-1,2,3-triazol-4-yl]acetamide (9b)

[3-(Pivaloyloxymethyl)-5-(perylen-3-ylethynyl)uracil-1-yl]-*N*-[1-(2-hydroxyethyl)-1,2,3-triazol-4-yl]acetamide (9b) was prepared using 2-azidoethanol. Gradient elution with MeOH (1 → 6%) in CHCl_3_ and evaporation gave pure compound as orange solid, yield 120 mg (67%). *R*_f_ 0.6 (10% MeOH in CHCl_3_); UV-Vis (20% DMSO in 96% EtOH) *λ*_max_, nm (*ε*, M^−1^ cm^−1^): 441 (38 600), 470 (48 000), *λ*_min_, nm (*ε*, M^−1^ cm^−1^): 452 (26 800). Fluorescence (96% EtOH, *λ*_em_ 530 nm, excitation *λ*_max_, nm): 334, 440, 468; (96% EtOH, *λ*_ex_ 420 nm, emission *λ*_max_, nm): 476, 509. ^1^H NMR (500 MHz, DMSO-*d*_6_) *δ* 8.85–8.8 (m, 1H), 8.47–8.43 (m, 2H), 8.42–8.33 (m, 3H), 8.27 (t, 1H, *J* = 8.23 Hz), 7.93 (s, 1H), 7.86–7.8 (m, 2H), 7.72–7.66 (m, 2H), 7.56 (t, 2H, *J* = 7.82 Hz), 5.86 (s, 2H), 4.54 (s, 2H), 4.41–4.36 (m, 4H), 3.78 (t, 2H, *J* = 4.8 Hz), 1.14 (s, 9H). ^13^C NMR (125 MHz, DMSO-*d*_6_) *δ* 176.6, 166.1, 160.5, 149.7, 149.5, 143.9, 134.2, 133.7, 131.3, 131.1, 130.5, 130.1, 129.8, 128.7, 128.4, 127.9, 127.8, 127.6, 127.0, 127.0, 125.6, 123.4, 121.7, 121.5, 121.3, 120.3, 119.2, 96.7, 90.9, 87.9, 65.1, 59.9, 52.2, 50.9, 38.3, 34.4, 26.7; HRMS (ESI, *m*/*z*): calcd for C_39_H_34_N_6_O_6_Na ([M + Na]^+^): 705.2432, found: 705.2422.

#### [3-(Pivaloyloxymethyl)-5-(perylen-3-ylethynyl)uracil-1-yl]-*N*-(1-{1,1,1-[tris(5-hydroxy-2-oxopentyl)]-3-oxahex-6-yl}-1,2,3-triazol-4-yl)acetamide (9c)

[3-(Pivaloyloxymethyl)-5-(perylen-3-ylethynyl)uracil-1-yl]-*N*-(1-{1,1,1-[tris(5-hydroxy-2-oxopentyl)]-3-oxahex-6-yl}-1,2,3-triazol-4-yl)acetamide (9c) was prepared using azide 2. Gradient elution with EtOH (1 → 50%) in CH_2_Cl_2_ and evaporation gave pure compound as dark brown solid, yield 210 mg (81%). *R*_f_ 0.27 (8% EtOH in CH_2_Cl_2_); UV-Vis (20% DMSO in 96% EtOH) *λ*_max_, nm (*ε*, M^−1^ cm^−1^): 441 (38 600), 470 (48 000), *λ*_min_, nm (*ε*, M^−1^ cm^−1^): 452 (26 800). Fluorescence (96% EtOH, *λ*_em_ 530 nm, excitation *λ*_max_, nm): 334, 440, 468; (96% EtOH, *λ*_ex_ 420 nm, emission *λ*_max_, nm): 476, 509. ^1^H NMR (400 MHz; DMSO-*d*_6_) *δ* 8.78 (t, 1H, *J* = 5.48 Hz), 8.48–8.26 (m, 6H), 7.94 (s, 1H), 7.86–7.8 (m, 2H), 7.73–7.65 (m, 2H), 7.57 (t, 2H, *J* = 7.87 Hz), 5.87 (s, 2H), 4.54 (s, 2H), 4.42–4.35 (m, 4H), 4.32 (t, 3H, *J* = 5.09 Hz), 3.48–3.41 (m, 6H), 3.39 (t, 6H, *J* = 6.36 Hz), 3.34 (t, 2H, *J* = 6.04 Hz), 3.3–3.25 (m, 8H), 2.07–1.99 (m, 2H), 1.62 (quint, 6H, *J* = 6.36 Hz), 1.14 (s, 9H). ^13^C NMR (100 MHz, DMSO-*d*_6_) *δ* 176.5, 166.0, 160.4, 149.7, 149.3, 144.0, 134.1, 133.7, 131.3, 131.0, 130.5, 130.0, 129.8, 128.6, 128.3, 127.8, 127.8, 127.6, 127.0, 126.9, 125.6, 122.9, 121.6, 121.4, 121.3, 120.3, 119.1, 96.7, 90.8, 87.9, 69.3, 69.1, 67.9, 67.3, 65.0, 57.9, 50.9, 46.6, 44.9, 38.3, 34.4, 32.6, 29.9, 26.6. HRMS (ESI, *m*/*z*): calcd for C_54_H_64_N_6_O_12_Na ([M + Na]^+^): 1011.4474, found: 1011.4458.

### Click reaction between alkyne 7 and polyazides 3–5, general procedure

Azide (0.025 mmol) and propargyl amide 7 with 5% excess for each azido group (0.53, 0.79 and 1.05 mmol respectively for cases 9d, 9e, 9f) were dissolved in DMSO (3 mL) under vigorous magnetic stirring. The solution was flushed with argon for degassing and 0.1 M solution of CuSO_4_·5H_2_O and TBTA ligand (1 : 1) in 55% DMSO were added (80 μL). Then ascorbic acid (100 mg mL^−1^ in H_2_O; 30 μL) was quickly loaded and the flask was tightly closed. After 12 h the reaction mixture was quenched with brine (6 mL) and the solid precipitated was isolated using centrifugation. The brine was decanted and the crude product was washed with distilled water (2 × 3 mL). The residue was applied on the silica gel by evaporation of the acetone solution with small portion of the sorbent. The obtained powder was carefully spilled on the column filled with silica gel in CH_2_Cl_2_. The following compounds were obtained.

#### 1,1-[Bis(5-hydroxy-2-oxapentyl)]-1,1-{bis[5-(4-{*N*-[(3-(pivaloyloxymethyl)-5-(perylen-3-ylethynyl)uracil-1-yl)acetyl]amino}-1,2,3-triazol-1-yl)-2-oxapent-1-yl]}methane (9d)

1,1-[Bis(5-hydroxy-2-oxapentyl)]-1,1-{bis[5-(4-{*N*-[(3-(pivaloyloxymethyl)-5-(perylen-3-ylethynyl)uracil-1-yl)acetyl]amino}-1,2,3-triazol-1-yl)-2-oxapent-1-yl]}methane (9d) was prepared using azide 3. Gradient elution with EtOH (1 → 30%) in CH_2_Cl_2_ and evaporation gave pure compound as orange solid, yield 28 mg (70%). *R*_f_ 0.34 (8% EtOH in CH_2_Cl_2_); UV-Vis (20% DMSO in 96% EtOH) *λ*_max_, nm (*ε*, M^−1^ cm^−1^): 442 (86 000), 470 (96 100), *λ*_min_, nm (*ε*, M^−1^ cm^−1^): 454 (66 800). Fluorescence (96% EtOH, *λ*_em_ 530 nm, excitation *λ*_max_, nm): 336, 441, 469; (96% EtOH, *λ*_ex_ 420 nm, emission *λ*_max_, nm): 477, 510. ^1^H NMR (400 MHz, DMSO-*d*_6_) *δ* 8.78 (t, 2H, *J* = 5.48 Hz), 8.43–8.22 (m, 12H), 7.93 (s, 2H), 7.83–7.77 (m, 4H), 7.7–7.62 (m, 4H), 7.58–7.5 (m, 4H), 5.85 (s, 4H), 4.54 (s, 4H), 4.41–4.31 (m, 10H), 3.48–3.3 (m, 12H), 3.3–3.25 (m, 8H), 2.07–1.98 (m, 4H), 1.66–1.58 (m, 4H), 1.12 (s, 18H). ^13^C NMR (100 MHz, DMSO-*d*_6_) *δ* 176.6, 166.1, 160.5, 149.7, 149.3, 144.1, 134.2, 133.7, 131.3, 131.0, 130.5, 130.1, 129.8, 128.7, 128.3, 127.9, 127.8, 127.6, 127.0, 126.9, 125.6, 122.9, 121.6, 121.4, 121.3, 120.3, 119.1, 96.8, 90.9, 87.8, 69.2, 69.0, 68.0, 67.4, 65.1, 57.9, 51.0, 46.7, 45.0, 38.3, 34.4, 32.6, 29.9, 26.6. HRMS (ESI, *m*/*z*): calcd for C_91_H_93_N_12_O_16_ ([M + H]^+^): 1609.6827, found: 1609.6813.

#### 1-(5-Hydroxy-2-oxapentyl)-1,1,1-{tris[5-(4-{*N*-[(3-(pivaloyloxymethyl)-5-(perylen-3-ylethynyl)uracil-1-yl)acetyl]amino}triazol-1-yl)-2-oxapent-1-yl]}methane (9e)

1-(5-Hydroxy-2-oxapentyl)-1,1,1-{tris[5-(4-{*N*-[(3-(pivaloyloxymethyl)-5-(perylen-3-ylethynyl)uracil-1-yl)acetyl]amino}triazol-1-yl)-2-oxapent-1-yl]}methane (9e) was prepared using azide 4. Gradient elution with EtOH (1 → 20%) in CH_2_Cl_2_ and evaporation gave pure compound as orange solid, yield 30 mg (54%). *R*_f_ 0.39 (8% EtOH in CH_2_Cl_2_); UV-Vis (20% DMSO in 96% EtOH) *λ*_max_, nm (*ε*, M^−1^ cm^−1^): 443 (114 700), 471 (124 700), *λ*_min_, nm (*ε*, M^−1^ cm^−1^): 455 (91 300). Fluorescence (96% EtOH, *λ*_em_ 530 nm, excitation *λ*_max_, nm): 335, 440, 468; (96% EtOH, *λ*_ex_ 420 nm, emission *λ*_max_, nm): 477, 511. ^1^H NMR (400 MHz, DMSO-*d*_6_) *δ* 8.77 (t, 3H, *J* = 5.48 Hz), 8.43–8.22 (m, 18H), 7.93 (s, 3H), 7.83–7.77 (m, 6H), 7.68–7.62 (m, 6H), 7.57–7.5 (m, 6H), 5.85 (s, 6H), 4.53 (s, 6H), 4.4–4.34 (m, 12H), 4.32 (t, 1H, *J* = 5.17 Hz), 3.43–3.34 (m, 10H), 3.3–3.26 (m, 8H), 2.06–1.97 (m, 6H), 1.65–1.57 (m, 2H), 1.12 (s, 27H). ^13^C NMR (100 MHz, DMSO-*d*_6_) *δ* 176.5, 166.1, 160.4, 149.7, 149.3, 144.1, 134.1, 133.7, 131.3, 131.0, 130.5, 130.0, 129.7, 129.5, 128.6, 128.3, 127.8, 127.7, 127.6, 127.0, 126.9, 125.5, 122.9, 121.6, 121.3, 121.2, 120.2, 119.1, 96.8, 90.9, 87.8, 69.1, 68.0, 67.4, 65.0, 57.9, 50.9, 46.6, 44.9, 38.3, 34.4, 32.6, 29.9, 26.6. HRMS (ESI, *m*/*z*): calcd for C_128_H_120_N_18_O_20_Na ([M + Na]^+^): 2251.8818, found: 2251.8766.

#### Tetrakis[5-(4-{*N*-[(3-(pivaloyloxymethyl)-5-(perylen-3-ylethynyl)uracil-1-yl)acetyl]amino}triazol-1-yl)-2-oxapent-1-yl]methane (9f)

Tetrakis[5-(4-{*N*-[(3-(pivaloyloxymethyl)-5-(perylen-3-ylethynyl)uracil-1-yl)acetyl]amino}triazol-1-yl)-2-oxapent-1-yl]methane (9f) was prepared using azide 5. Gradient elution with EtOH (1 → 25%) in CH_2_Cl_2_ and evaporation gave pure compound as orange solid, yield 19 mg (38%). *R*_f_ 0.44 (8% EtOH in CH_2_Cl_2_); UV-Vis (20% DMSO in 96% EtOH) *λ*_max_, nm (*ε*, M^−1^ cm^−1^): 443 (131 500), 471 (138 300), *λ*_min_, nm (*ε*, M^−1^ cm^−1^): 455 (107 200). Fluorescence (96% EtOH, *λ*_em_ 530 nm, excitation *λ*_max_, nm): 419, 440, 464; (96% EtOH, *λ*_ex_ 420 nm, emission *λ*_max_, nm): 452, 477, 509. ^1^H NMR (400 MHz, DMSO-*d*_6_) *δ* 8.79–8.75 (m, 4H), 8.41–8.21 (m, 24H), 7.93 (s, 4H), 7.81–7.76 (m, 8H), 7.66–7.6 (m, 8H), 7.55–7.48 (m, 8H), 5.84 (s, 8H), 4.53 (s, 8H), 4.4–4.33 (m, 16H), 3.38–3.32 (m, 8H), 3.28 (s, 8H), 2.06–1.97 (m, 8H), 1.11 (s, 36H). ^13^C NMR (100 MHz, DMSO-*d*_6_) *δ* 176.5, 166.0, 160.4, 149.7, 149.2, 144.1, 134.1, 133.7, 131.2, 131.0, 130.4, 130.0, 129.7, 128.6, 128.3, 127.8, 127.7, 127.5, 126.9, 126.9, 125.5, 122.9, 121.5, 121.3, 121.2, 120.2, 119.1, 96.8, 90.9, 87.8, 68.9, 67.3, 65.0, 50.9, 46.6, 38.2, 34.4, 29.8, 28.9, 26.6. HRMS (ESI, *m*/*z*): calcd for C_165_H_148_N_24_O_24_Na ([M + Na]^+^): 2872.0991, found: 2872.0181.

### Pom group removal, a general procedure

Pom-protected compound (66 μmol) was dissolved in the mixture containing DMSO (1.2 mL) and methanol (600 μL). To this solution 10 M NaOH (60 μL) was added under vigorous stirring. After 30 min TLC showed full consumption of the starting material. The reaction mixture was poured into 10% citric acid (6 mL). The precipitate was centrifuged thoroughly and washed with water (4 × 3 mL). The residue was applied on the silica gel using evaporation of the acetone solution with small portion of the sorbent. The obtained powder was carefully spilled on the column filled with silica gel in CHCl_3_. Elution with gradient of EtOH in CHCl_3_ from 5 to 30% and evaporation gave pure compound as orange solid. The following compounds were isolated.

#### [5-(Perylen-3-ylethynyl)uracil-1-yl]-*N*-propargylacetamide (8)

Yield 13 mg (41%). *R*_f_ 0.50 (5% MeOH in CHCl_3_); UV-Vis (20% DMSO in 96% EtOH) *λ*_max_, nm (*ε*, M^−1^ cm^−1^): 440 (38 800), 468 (47 520), *λ*_min_, nm (*ε*, M^−1^ cm^−1^): 450 (28 200). Fluorescence (96% EtOH, *λ*_em_ 530 nm, excitation *λ*_max_, nm): 440, 466; (96% EtOH, *λ*_ex_ 420 nm, emission *λ*_max_, nm): 450, 475, 508. ^1^H NMR (500 MHz, DMSO-*d*_6_) *δ* 11.83 (br s, 1H), 8.82–8.78 (m, 1H), 8.43 (d, 1H, *J* = 7.32 Hz), 8.4–8.3 (m, 3H), 8.27 (d, 1H, *J* = 8.24 Hz), 7.84–7.79 (m, 2H), 7.71–7.63 (m, 2H), 7.58–7.52 (m, 2H), 7.47 (s, 2H), 3.97–3.91 (m, 2H), 3.18 (t, 1H, *J* = 2.44 Hz). ^13^C NMR (100 MHz, DMSO-*d*_6_) *δ* 166.4, 162.1, 150.0, 149.9, 134.2, 133.7, 131.1, 131.0, 130.4, 130.1, 129.8, 128.6, 128.3, 127.8, 127.7, 127.6, 127.1, 127.0, 125.7, 121.6, 121.4, 121.3, 120.3, 119.5, 97.4, 90.6, 88.6, 80.7, 73.5, 49.8, 28.1. HRMS (ESI, *m*/*z*): calcd for C_31_H_20_N_3_O_3_ ([M + H]^+^): 482.1499, found: 482.1489.

#### [5-(Perylen-3-ylethynyl)uracil-1-yl]-*N*-(1-benzyl-1,2,3-triazol-4-yl)acetamide (10a)

Yield 21 mg (35%). *R*_f_ 0.36 (10% EtOH in CHCl_3_) UV-Vis (20% DMSO in 96% EtOH) *λ*_max_, nm (*ε*, M^−1^ cm^−1^): 440 (38 800), 468 (47 520), *λ*_min_, nm (*ε*, M^−1^ cm^−1^): 450 (28 200). Fluorescence (96% EtOH, *λ*_em_ 530 nm, excitation *λ*_max_, nm): 440, 466; (96% EtOH, *λ*_ex_ 420 nm, emission *λ*_max_, nm): 475, 508. ^1^H NMR (400 MHz, DMSO-*d*_6_) *δ* 11.78 (br s, 1H), 8.73 (t, 1H, *J* = 5.48 Hz), 8.45 (d, 1H, *J* = 7.47 Hz), 8.43–8.33 (m, 3H), 8.31–8.26 (m, 2H), 8.0 (s, 1H), 7.87–7.81 (m, 2H), 7.73–7.65 (m, 2H), 7.57 (t, 2H, *J* = 7.79 Hz), 7.41–7.28 (m, 5H), 5.58 (s, 2H), 4.45 (s, 2H), 4.37 (d, 2H, *J* = 5.56 Hz). ^13^C NMR (100 MHz, DMSO-*d*_6_) *δ* 166.4, 162.1, 150.0, 149.8, 144.5, 136.0, 134.2, 133.7, 131.1, 131.0, 130.4, 130.1, 129.8, 128.7, 128.6, 128.3, 128.1, 127.9, 127.8 (two overlapped signals), 127.6, 127.0, 127.0, 125.7, 123.1, 121.6, 121.4, 121.3, 120.3, 119.5, 97.3, 90.5, 88.6, 52.7, 49.9, 34.3. HRMS (ESI, *m*/*z*): calcd for C_38_H_27_N_6_O_3_ ([M + H]^+^): 615.2139, found: 615.2135.

#### [5-(Perylen-3-ylethynyl)uracil-1-yl]-*N*-[1-(2-hydroxyethyl)-1,2,3-triazol-4-yl]acetamide (10b)

Yield 15 mg (40%). *R*_f_ 0.44 (25% EtOH in CHCl_3_) UV-Vis (20% DMSO in 96% EtOH) *λ*_max_, nm (*ε*, M^−1^ cm^−1^): 441 (53 600), 470 (66 000), *λ*_min_, nm (*ε*, M^−1^ cm^−1^): 452 (37 600). Fluorescence (96% EtOH, *λ*_em_ 530 nm, excitation *λ*_max_, nm): 440, 466; (96% EtOH, *λ*_ex_ 420 nm, emission *λ*_max_, nm): 475, 508. ^1^H NMR (400 MHz, DMSO-*d*_6_) *δ* 11.79 (br s, 1H), 8.75 (t, 1H, *J* = 5.64 Hz), 8.45 (d, 1H, *J* = 7.47 Hz), 8.43–8.33 (m, 3H), 8.33–8.25 (m, 2H), 7.91 (s, 1H), 7.87–7.8 (m, 2H), 7.74–7.64 (m, 2H), 7.57 (t, 2H, *J* = 7.47 Hz), 5.01 (t, 1H, *J* = 5.25 Hz), 4.47 (s, 2H), 4.42–4.34 (m, 4H), 3.78 (q, 2H, *J* = 5.35 Hz). ^13^C NMR (100 MHz, DMSO-*d*_6_) *δ* 166.4, 162.1, 150.0, 149.9, 144.0, 134.2, 133.7, 131.1, 131.0, 130.4, 130.1, 129.8, 128.6, 128.3, 127.8, 127.8, 127.6, 127.0, 127.0, 125.7, 123.3, 121.6, 121.4, 121.3, 120.3, 119.5, 97.3, 90.5, 88.6, 59.8, 52.1, 49.9, 34.3. HRMS (ESI, *m*/*z*): calcd for C_33_H_25_N_6_O_4_ ([M + H]^+^): 569.1932, found: 569.1926.

#### [5-(Perylen-3-ylethynyl)uracil-1-yl]-*N*-(1-{1,1,1-[tris(5-hydroxy-2-oxapentyl)]-3-oxahex-6-yl}-1,2,3-triazol-4-yl)acetamide (10c)

Yield 18 mg (30%). *R*_f_ 0.56 (25% MeOH in CHCl_3_) UV-Vis (20% DMSO in 96% EtOH) *λ*_max_, nm (*ε*, M^−1^ cm^−1^): 440 (38 800), 468 (47 520), *λ*_min_, nm (*ε*, M^−1^ cm^−1^): 450 (28 200). Fluorescence (96% EtOH, *λ*_em_ 530 nm, excitation *λ*_max_, nm): 440, 467; (96% EtOH, *λ*_ex_ 420 nm, emission *λ*_max_, nm): 476, 508. ^1^H NMR (400 MHz, DMSO-*d*_6_) *δ* 11.78 (br s, 1H), 8.73 (t, 1H, *J* = 5.48 Hz), 8.45 (d, 1H, *J* = 7.47 Hz), 8.43–8.33 (m, 3H), 8.32–8.26 (m, 2H), 7.94 (s, 1H), 7.87–7.81 (m, 2H), 7.72–7.65 (m, 2H), 7.57 (t, 2H, *J* = 7.63 Hz), 4.46 (s, 2H), 4.41–4.35 (m, 4H), 4.32 (t, 3H, *J* = 5.17 Hz), 3.48–3.41 (m, 6H), 3.38 (t, 6H, *J* = 6.36 Hz), 3.36–3.31 (m, 2H), 3.3–3.24 (m, 8H), 2.07–1.98 (m, 2H), 1.62 (quint, 6H, *J* = 6.36 Hz). ^13^C NMR (100 MHz, DMSO-*d*_6_) *δ* 166.4, 162.1, 150.0, 149.8, 144.1, 134.2, 133.7, 131.1, 131.0, 130.4, 130.1, 129.8, 128.6, 128.3, 127.8, 127.6, 127.0, 126.9, 125.7, 122.9, 121.6, 121.4, 121.3, 120.3, 119.5, 97.3, 90.6, 88.6, 69.3, 69.1, 67.9, 67.3, 57.9, 49.9, 46.6, 44.9, 34.3, 32.6, 29.9. HRMS (ESI, *m*/*z*): calcd for C_48_H_55_N_6_O_10_ ([M + H]^+^): 875.3974, found: 875.3973.

### Cell toxicity assay

A cytotoxicity test in porcine embryo kidney (PEK) cells was performed as described previously.^2*a*,6,19,25^ In brief, PEK cells were seeded and incubated for 72 h at 37 °C. Stock solutions of the compounds with the concentration of 5 mM were prepared in 100% DMSO (Sigma). Two-fold dilutions of studied compounds were prepared in medium 199 on Earle solution to obtain final concentrations starting from 50 μM. Equal volumes of compound dilutions were added in four replicates to the cells. Cell control was treated with the same sequential concentrations of DMSO as in compounds dilutions in four replicates. After incubation at 37 °C on days 1 or 7 CC_50_ values were calculated according to the Kerber method.^28^

### Antiviral activity assays

Plaque reduction test was performed as previously described^2*a*,6,19,25^ on tick-borne encephalitis virus strain Absettarov. Stock solutions of the compounds with concentration of 5 mM were prepared in 100% DMSO (Sigma). Compounds were added to PEK cells simultaneously with virus and incubated at 37 °C for 1 h with gentle shaking every 15–20 min. Then, each well was overlaid with 1 mL of 1.26% methylcellulose (Sigma) containing 2% FBS (Gibco). After incubation at 37 °C in CO_2_ incubator for 6 days cells were fixed with 96% ethanol. Plaques were stained with 0.4% gentian violet. EC_50_ values were calculated according to Reed-and-Muench method.^29^

### Statistical analysis

Data are expressed as means ± standard deviations. The statistical significance was analyzed using Student's *t* test for at least three independent experiments.

### Molecular modelling

The molecules were drawn in InstantJChem^30^ and transferred to SYBYL-X 2.1 (ref. 27) in MOL format. MMFF94 charges^31^ were assigned to the atoms and the Powell^32^ optimization (10 000 steps, gradient termination, threshold 0.05 kcal (mol^−1^ × Å)) was performed in MMFF94s force field.^31*b*^ Then Confort^26^ global minimization was performed, sampling 2000 conformations (program maximum) and optimizing them with termination by negative change. Precision parameter was set to 0.001. Electrostatics was considered. The number of acyclic rotors concurrently sampled was set to 200, rotors in cycles were not sampled due to the aromaticity of all the studied cycles. Compound 9f contains more atoms than can be treated by Confort and thus was not optimized using this method. Fast Connolly surfaces^33^ for the optimized structures were calculated by MOLCAD in SYBYL-X 2.1 and colored according to hydrophobic potential.

## Conclusions

In summary, we used pentaerythritol-based azides for the preparation of clusters containing up to four residues of 5-(perylen-3-ylethynyl)uracil, an antiviral scaffold. UV-Vis, fluorescence, and anti-TBEV properties of compounds were studied. For the first time, the antiviral activity of RAFI clusters was demonstrated. Four compounds, including tetramer 9f, showed one-digit nanomolar activity against TBEV, being among the most potent anti-TBEV molecules to date. Some structural features of the most active compounds can be used in further design of antivirals.

## Conflicts of interest

There are no conflicts of interest to declare.

## Supplementary Material

RA-009-C9RA06313G-s001
