# Supplementary material for: Ramified derivatives of 5-(perylen-3-ylethynyl)uracil-1-acetic acid and their antiviral properties
Source: RSC Adv. 2019 Aug 20;9(45):26014–23. doi: 10.1039/c9ra06313g (PMC9070374; doi:10.1039/c9ra06313g)

Electronic Supplementary Material (ESI) for RSC Advances.
This journal is © The Royal Society of Chemistry 2019

Supporting Information

Ramified derivatives of 5-(perylen-3-ylethynyl)uracil-1-acetic acid and their antiviral properties

Ksenia A. Sapozhnikova,[a] Nikita A. Slesarchuk,[a,b] Alexey A. Orlov,[b,c,d] Evgeny V. Khvatov,[a,c] Eugene V. Radchenko,[b] Alexey A. Chistov,[a] Alexey V. Ustinov,[a,e,f] Vladimir A. Palyulin,[b] Liubov I. Kozlovskaya,[c,d] Dmitry I. Osolodkin,[b,c,d] Vladimir A. Korshun[a,f] and Vladimir A. Brylev[a,e]

[a.] *Shemyakin-Ovchinnikov Institute of Bioorganic Chemistry, Miklukho-Maklaya 16/10, Moscow 117997, Russia.*
[b.] *Department of Chemistry, Lomonosov Moscow State University, Moscow 119991, Russia.*
[c.] *FSBSI Chumakov FSC R&D IBP RAS, 8 bd 1 Poselok Instituta Poliomielita, Poselenie Moskovsky, Moscow 108819, Russia*
[d.] *Sechenov First Moscow State Medical University, Moscow 119991, Russia*
[e.] *Biotech Innovations Ltd, Leninskie Gory 1 bd 75, Moscow 119992, Russia*
[f.] *Department of Biology and Biotechnology, National Research University Higher School of Economics, Vavilova 7, Moscow 117312, Russia*

Contents

NMR spectra of synthesized compounds **3**

NMR spectra of 6,6-bis(5-Hydroxy-2-oxapentyl)-4,8-dioxa-11-hydroxyundec-1-yl azide (**2**)........ 3

NMR spectra of (3-(Pivaloyloxymethyl)-5-(perylen-3-ylethynyl)uracil-1)-N-propargyl acetamide (**7**)..... 4

NMR spectra of (3-(Pivaloyloxymethyl)-5-(perylen-3-ylethynyl)uracil-1)-N-(1-benzyltriazol-4-yl)acetamide (**9a**) 5

NMR spectra of 3-(Pivaloyloxymethyl)-5-(perylen-3-ylethynyl)uracil-1)-N-(1-hydroxyethyltriazol-4-yl)acetamide (**9b**) 6

NMR spectra of (3-(Pivaloyloxymethyl)-5-(perylen-3-ylethynyl)uracil-1)-N-(1-{1,1,1-[tris(5-hydroxy-2-oxapentyl)]-3-oxahex-6-yl}triazol-4-yl)acetamide (**9c**) 7

NMR spectra of 1,1-[bis(5-hydroxy-2-oxapentyl)]-1,1-{bis[5-(4-{N-[(3-(pivaloyloxymethyl)-5-(perylen-3-ylethynyl)uracil-1)acetyl]amino}triazol-1-yl)-2-oxapent-1-yl]} methane (**9d**) 8

NMR spectra of 1-(5-hydroxy-2-oxapentyl)-1,1,1-{tris[5-(4-{N-[(3-(Pivaloyloxymethyl)-5-(perylen-3-ylethynyl)uracil-1)acetyl] amino}triazol-1-yl)-2-oxapent-1-yl]} methane (**9e**) 9

NMR spectra of tetrakis [5-(4-{N-[(3-(Pivaloyloxymethyl)-5-(perylen-3-ylethynyl) uracil-1)acetyl]amino}triazol-1-yl)-2-oxapent-1-yl] methane (**9f**) 10

NMR spectra of (5-(Perylen-3-ylethynyl)uracil-1)-N-propargyl acetamide (**8**) 11

NMR spectra of 5-(perylen-3-ylethynyl)uracil-1)-N-(1-benzyltriazol-4-yl) acetamide (**10a**) 12

NMR spectra of 5-(perylen-3-ylethynyl) uracil-1)-N-(1-hydroxyethyltriazol-4-yl) acetamide (**10b**)....... 13

NMR spectra of 5-(perylen-3-ylethynyl) uracil-1)-N-(1-{1,1,1-[tris(5-hydroxy-2-oxapentyl)]-3-oxahex-6-yl} triazol-4-yl) acetamide (**10c**) 14

High resolution mass spectra of synthesized compounds **15**

(3-(Pivaloyloxymethyl)-5-(perylen-3-ylethynyl)uracil-1)-N-propargyl acetamide (**7**) 15

5-(Perylen-3-ylethynyl)uracil-1)-N-propargyl acetamide (**8**) 15

(3-(Pivaloyloxymethyl)-5-(perylen-3-ylethynyl)uracil-1)-N-(1-benzyltriazol-4-yl)acetamide (**9a**).......... 15

3-(Pivaloyloxymethyl)-5-(perylen-3-ylethynyl)uracil-1)-N-(1-hydroxyethyltriazol-4-yl)acetamide (**9b**) 15

(3-(Pivaloyloxymethyl)-5-(perylen-3-ylethynyl)uracil-1)-N-(1-{1,1,1-[tris(5-hydroxy-2-oxapentyl)]-3-oxahex-6-yl}triazol-4-yl)acetamide (**9c**) 15

1,1-[bis(5-hydroxy-2-oxapentyl)]-1,1-{bis[5-(4-{N-[(3-(pivaloyloxymethyl)-5-(perylen-3-ylethynyl)uracil-1)acetyl]amino}triazol-1-yl)-2-oxapent-1-yl]} methane (**9d**) 15

1-(5-hydroxy-2-oxapentyl)-1,1,1-{tris[5-(4-{N-[(3-(pivaloyloxymethyl)-5-(perylen-3-ylethynyl)uracil-1)acetyl] amino}triazol-1-yl)-2-oxapent-1-yl]} methane methane (**9e**) 16

tetrakis [5-(4-{N-[(3-(Pivaloyloxymethyl)-5-(perylen-3-ylethynyl) uracil-1)acetyl]amino}triazol-1-yl)-2-oxapent-1-yl] methane (**9f**) 16

5-(Perylen-3-ylethynyl)uracil-1)-N-(1-benzyltriazol-4-yl) acetamide (**10a**) 16

5-(Perylen-3-ylethynyl) uracil-1)-N-(1-hydroxyethyltriazol-4-yl) acetamide (**10b**) 16

5-(Perylen-3-ylethynyl) uracil-1)-N-(1-{1,1,1-[tris(5-hydroxy-2-oxapentyl)]-3-oxahex-6-yl} triazol-4-yl) acetamide (**10c**) 16

NMR spectra of synthesized compounds

NMR spectra of 6,6-bis(5-Hydroxy-2-oxapentyl)-4,8-dioxa-11-hydroxyundec-1-yl azide (**2**)

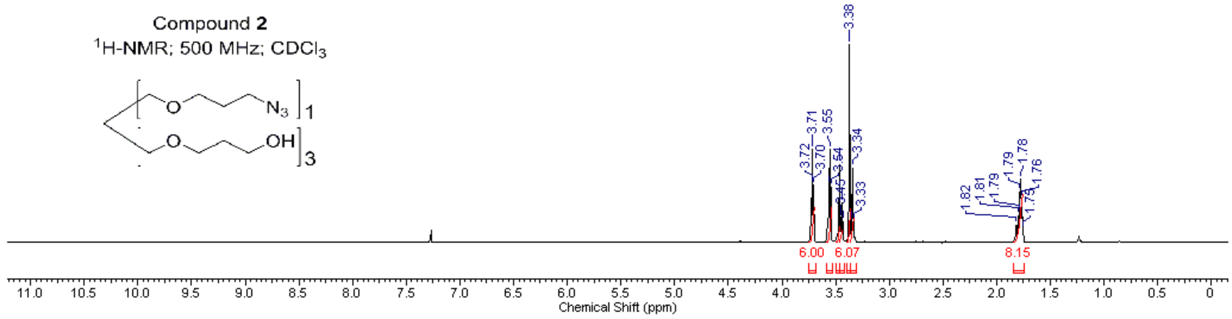

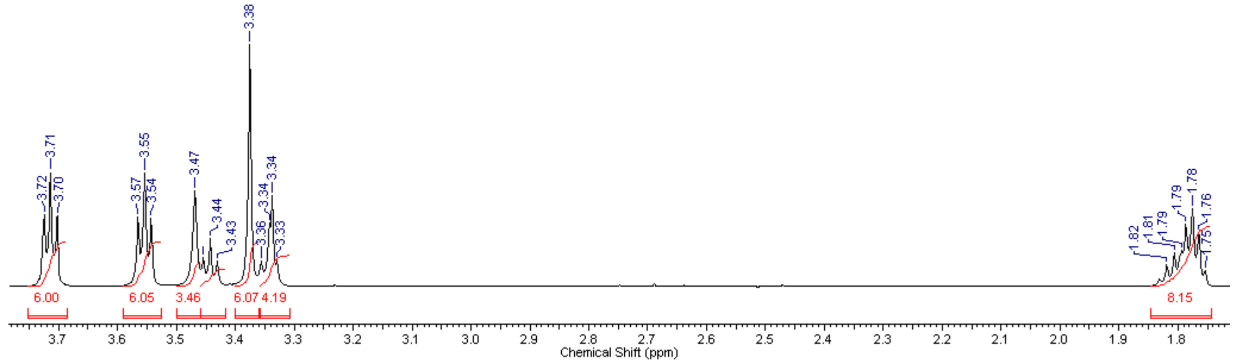

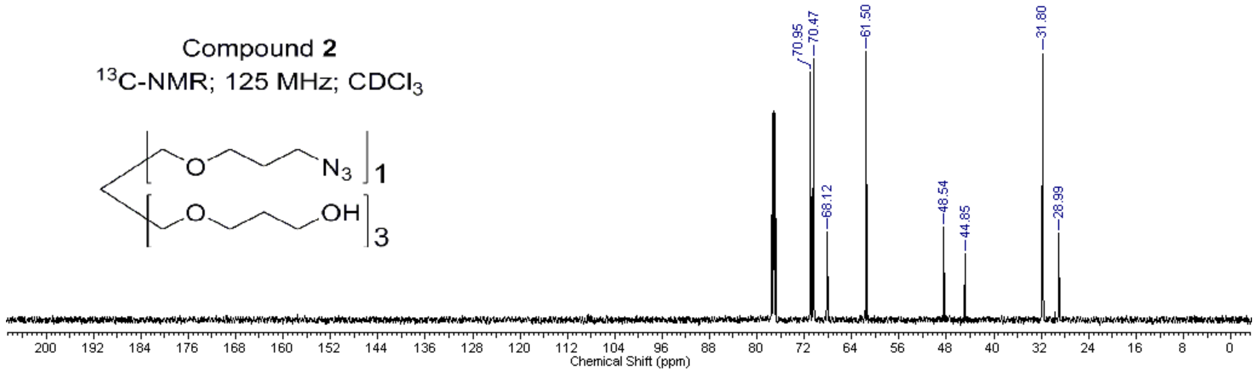

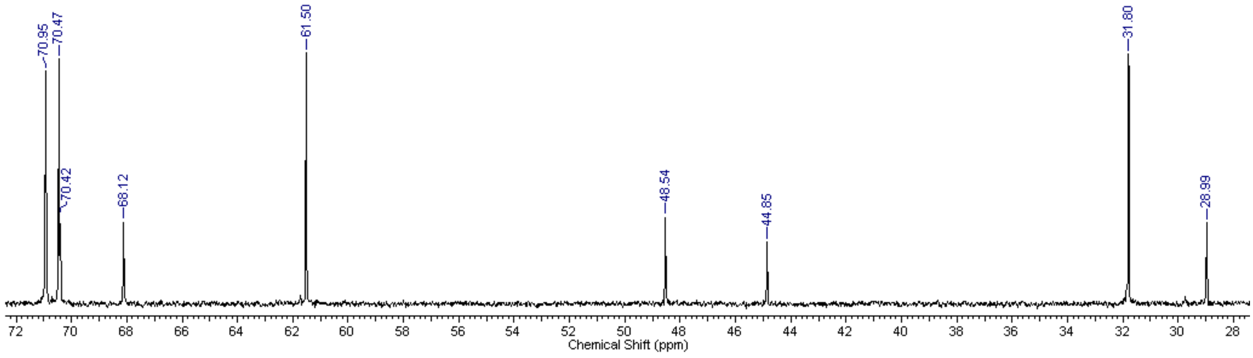

NMR spectra of (3-(Pivaloyloxymethyl)-5-(perylen-3-ylethynyl)uracil-1)-N-propargyl acetamide (7)

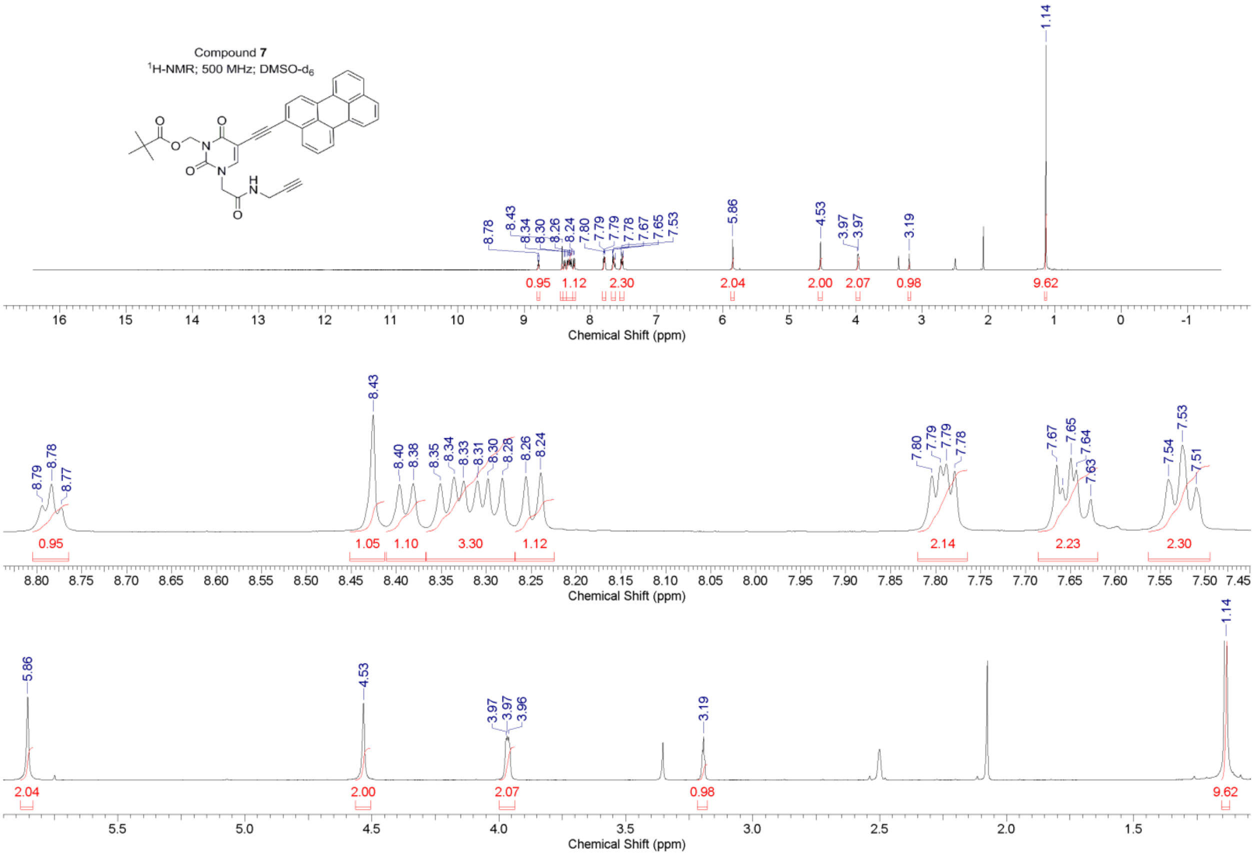

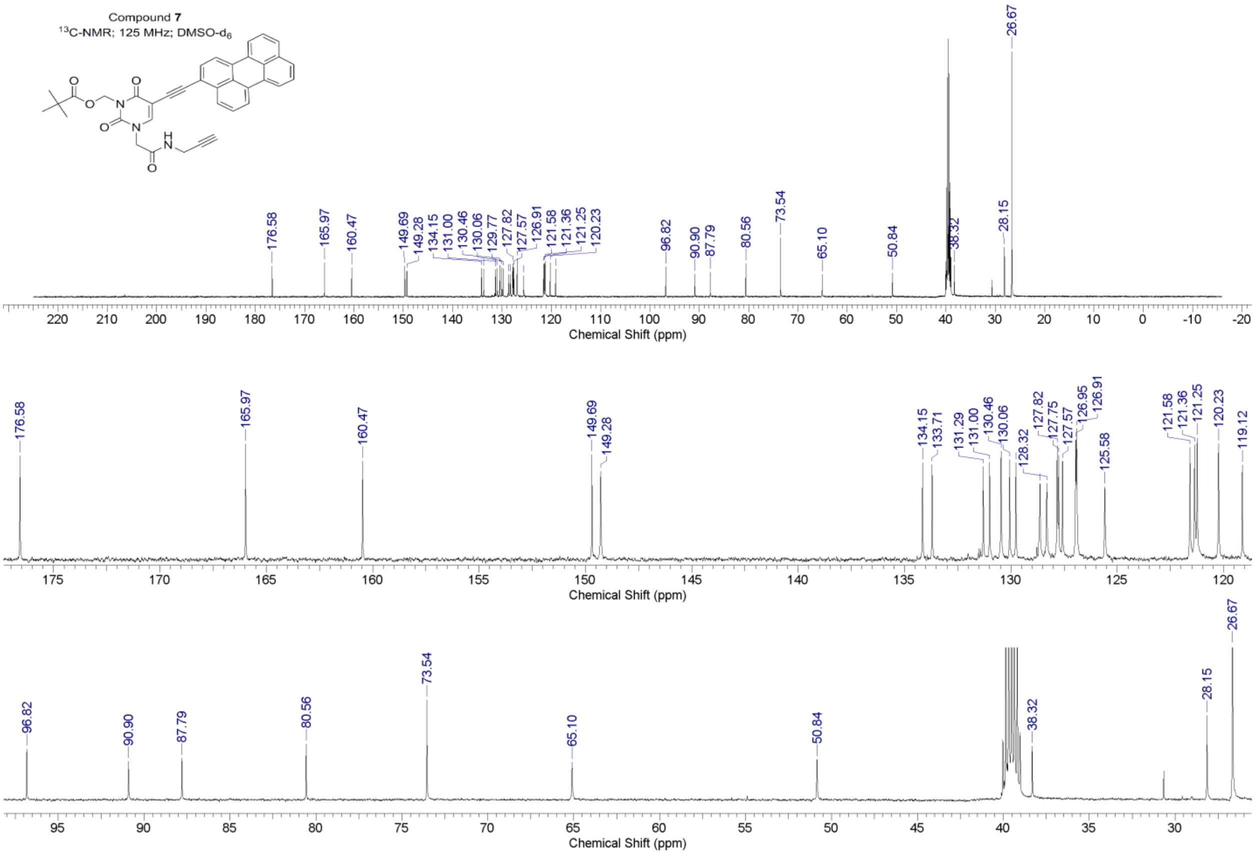

NMR spectra of (3-(Pivaloyloxymethyl)-5-(perylen-3-ylethynyl)uracil-1)-N-(1-benzyltriazol-4-yl)acetamide (**9a**)

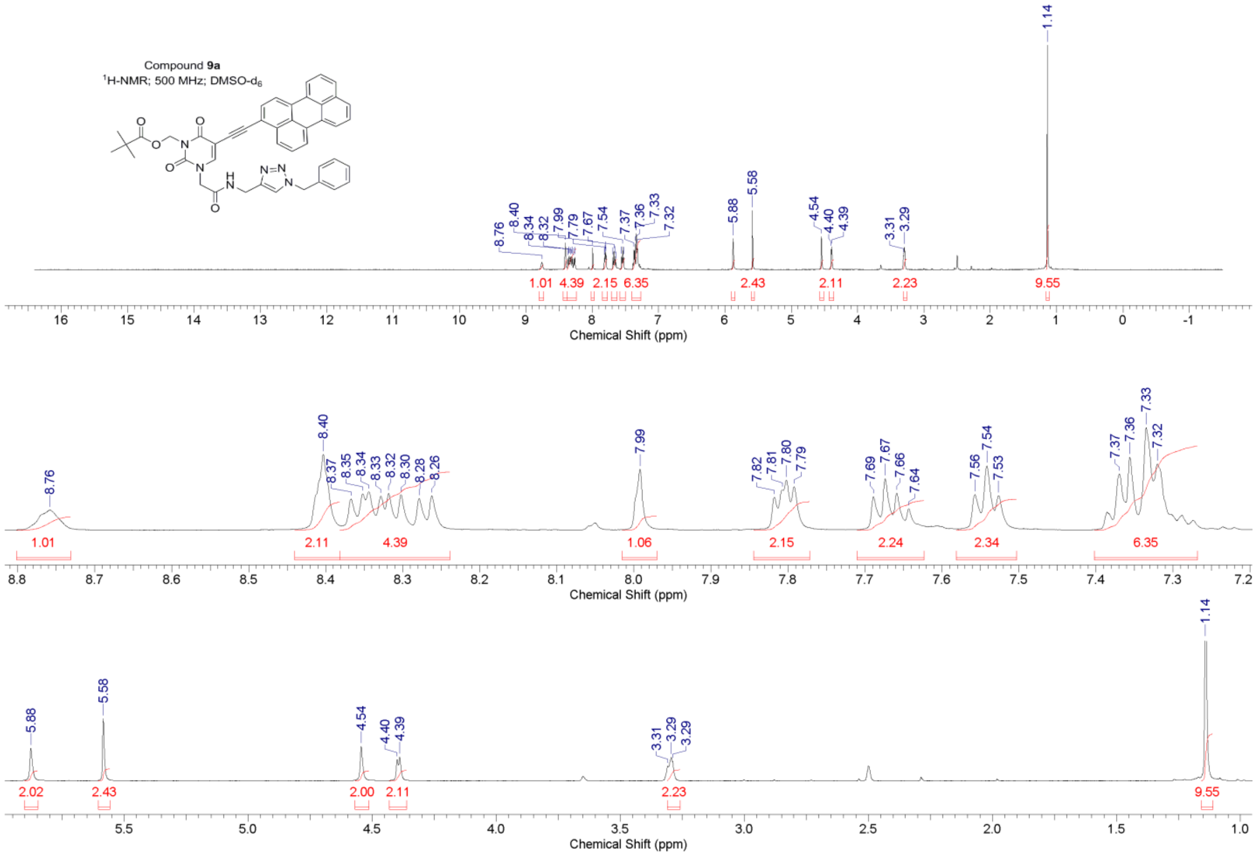

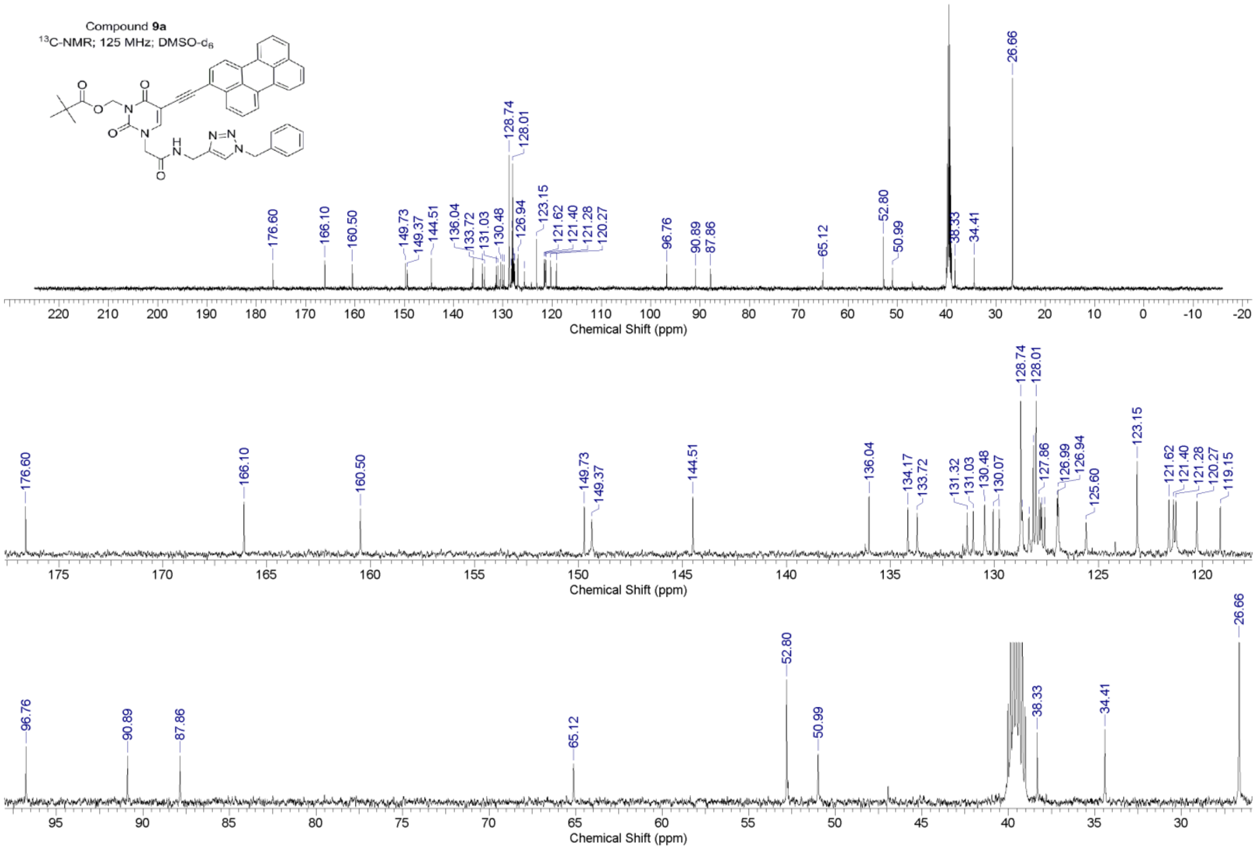

NMR spectra of 3-(Pivaloyloxymethyl)-5-(perylen-3-ylethynyl)uracil-1)-N-(1-hydroxyethyltriazol-4-yl)acetamide (**9b**)

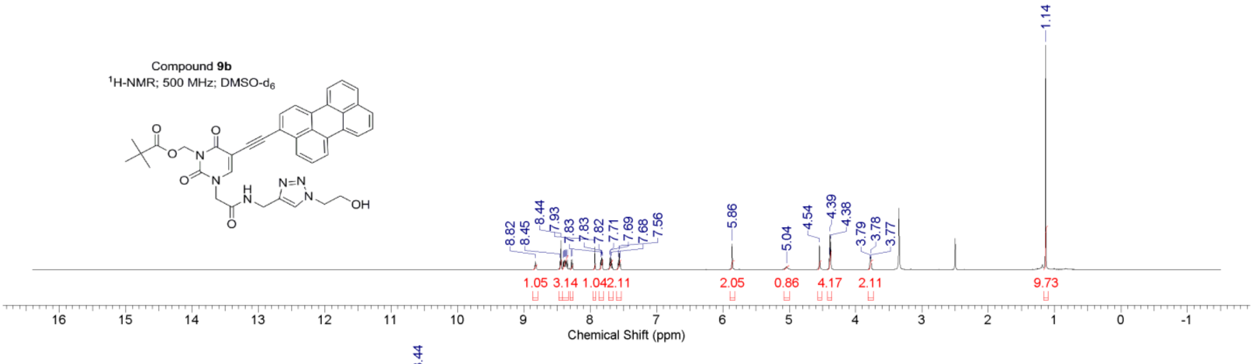

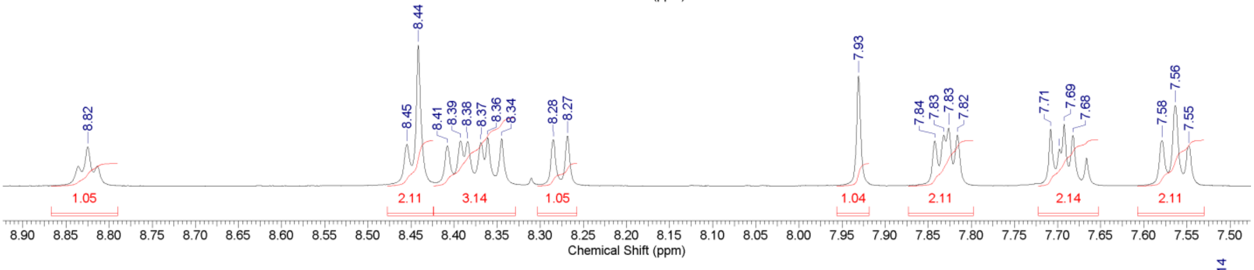

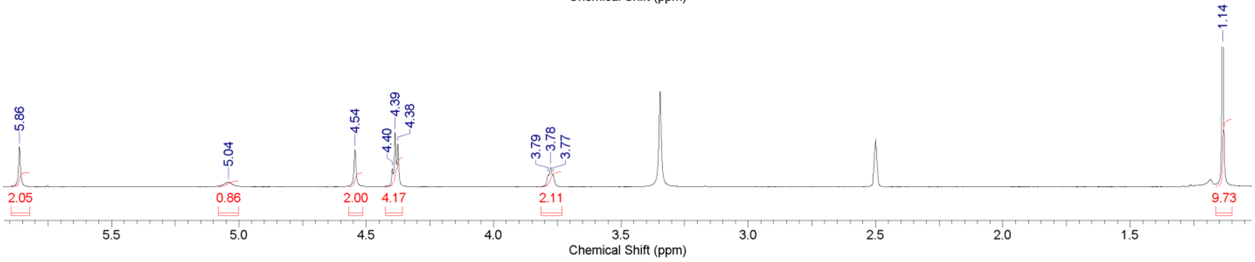

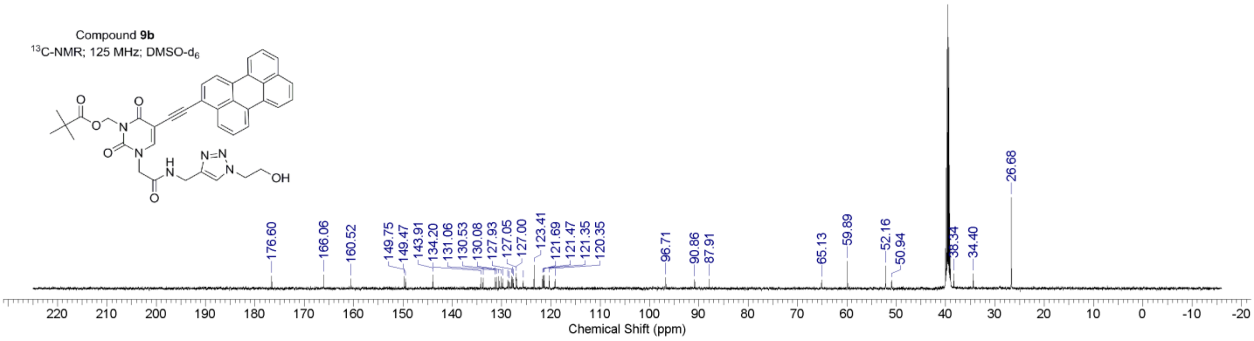

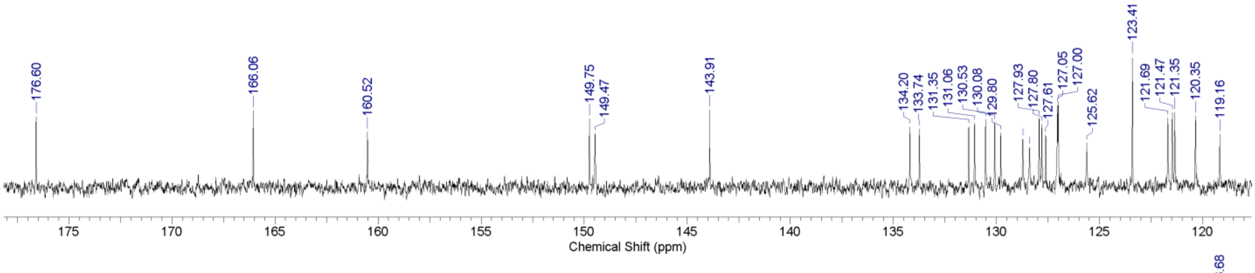

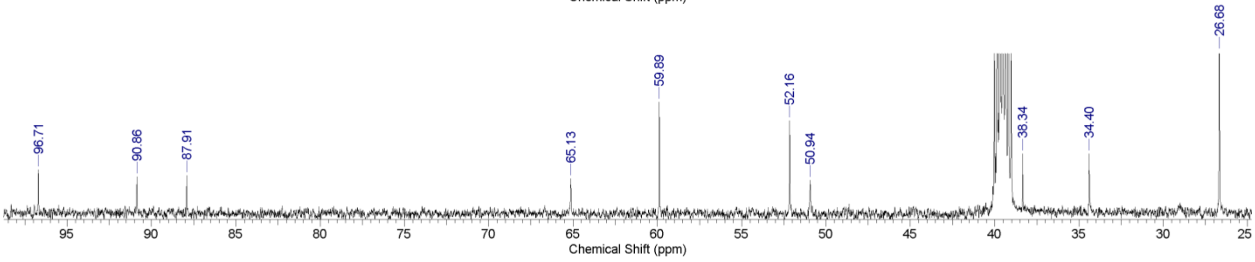

NMR spectra of (3-(Pivaloyloxymethyl)-5-(perylen-3-ylethynyl)uracil-1)-N-(1-{1,1,1-[tris(5-hydroxy-2-oxapentyl)]-3-oxahex-6-yl}triazol-4-yl)acetamide (**9c**)

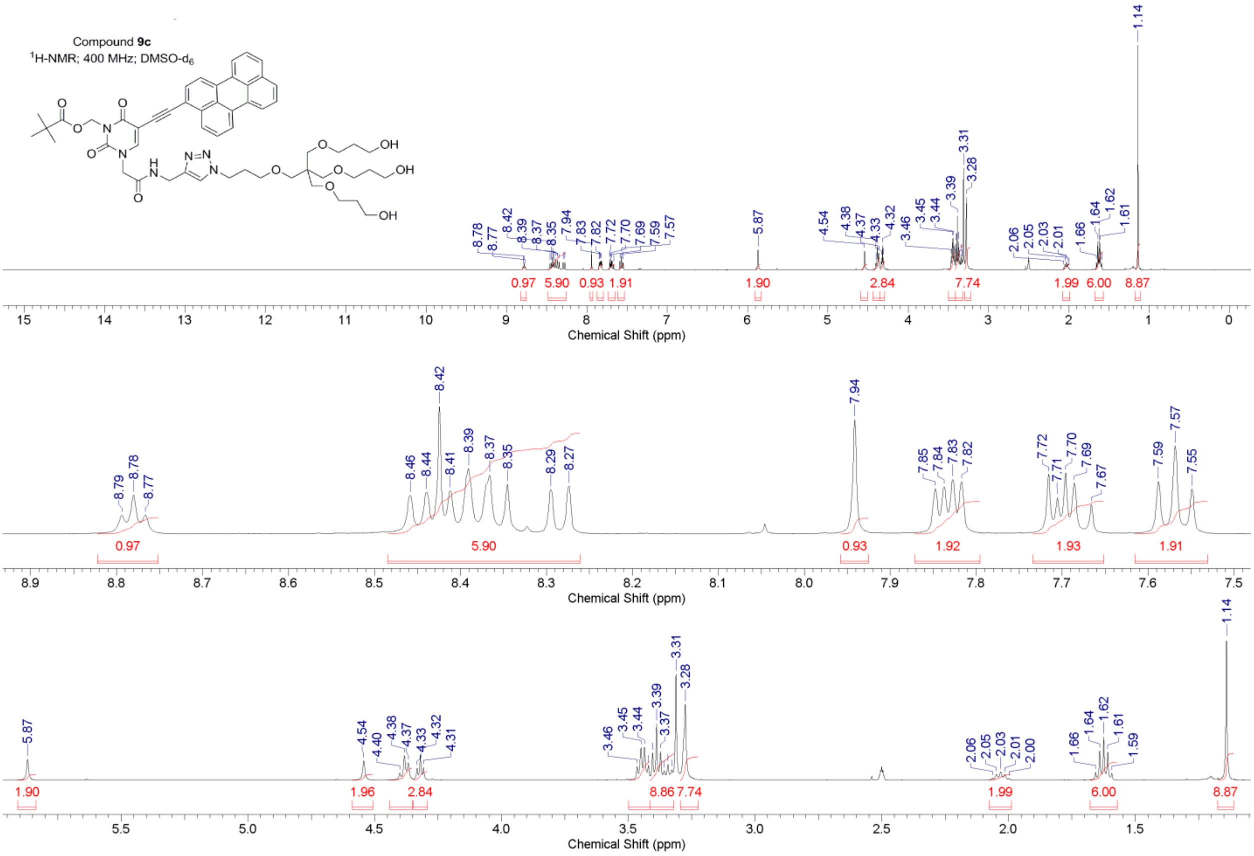

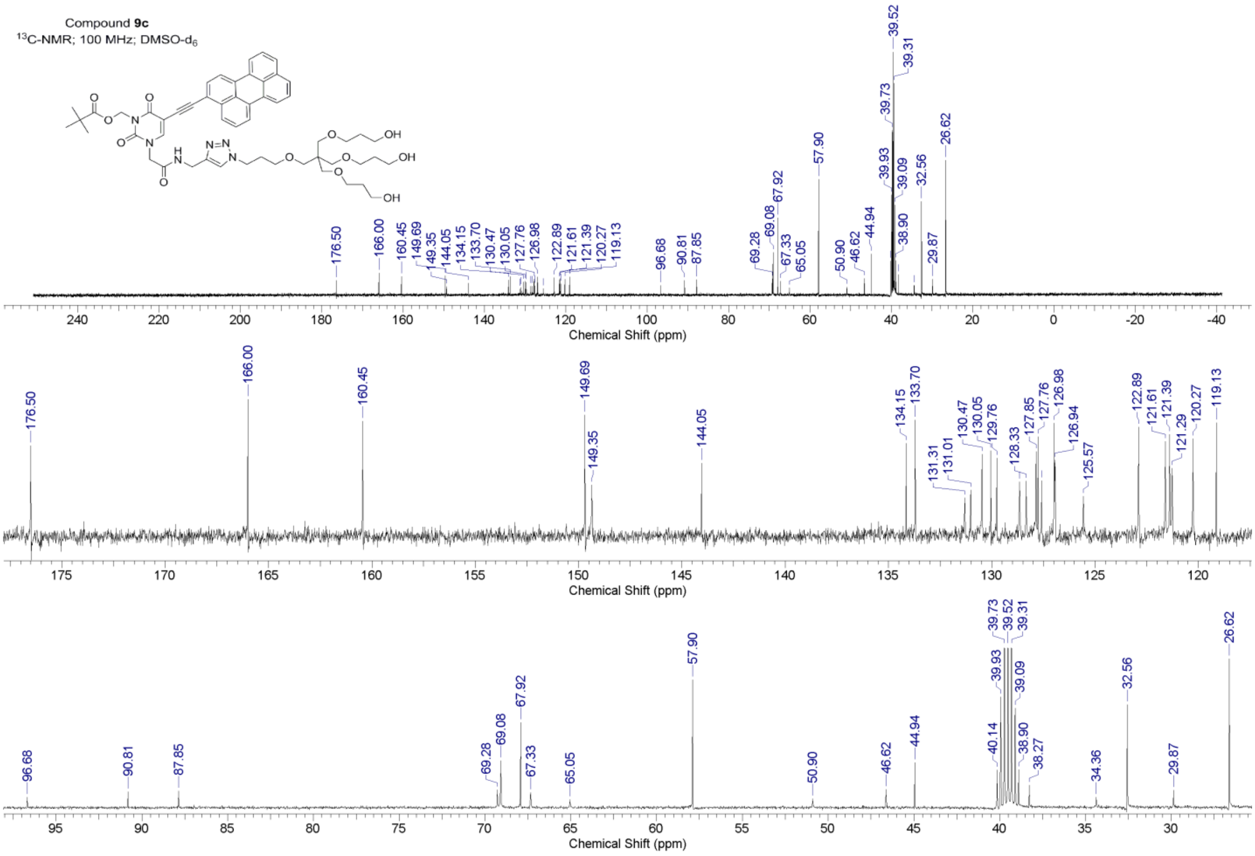

NMR spectra of 1,1-[bis(5-hydroxy-2-oxapentyl)]-1,1-{bis[5-(4-{N-[(3-(pivaloyloxymethyl)-5-(perylen-3-ylethynyl)uracil-1)acetyl]amino}triazol-1-yl)-2-oxapent-1-yl]} methane (**9d**)

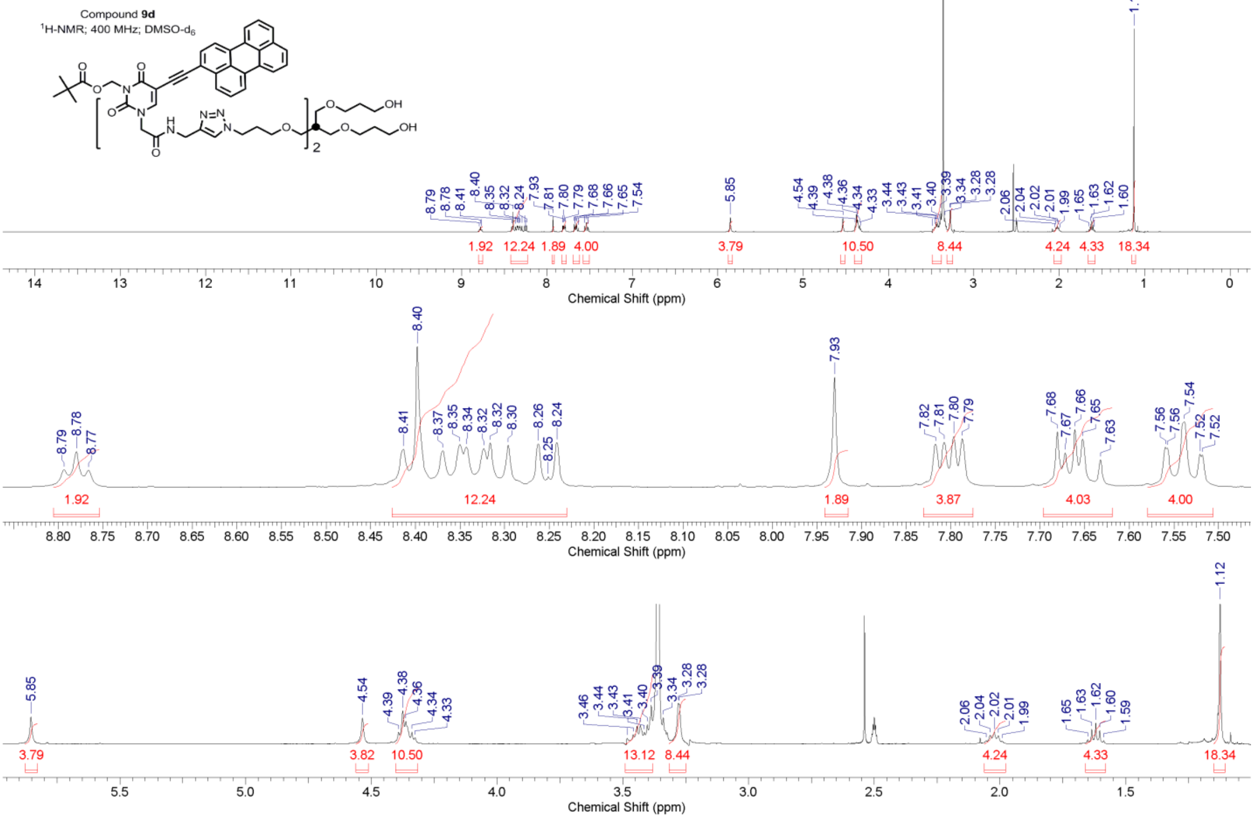

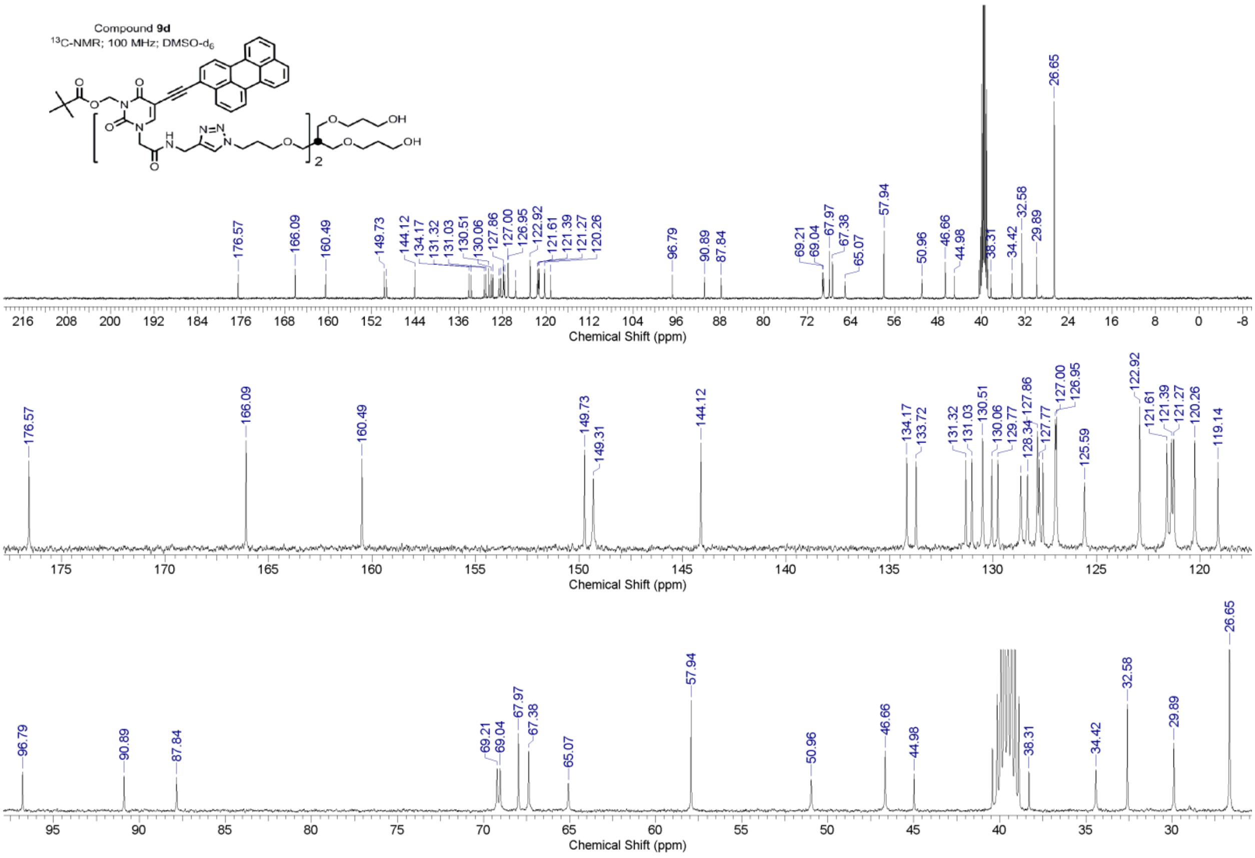

NMR spectra of 1-(5-hydroxy-2-oxapentyl)-1,1,1-{tris[5-(4-{N-[(3-(pivaloyloxymethyl)-5-(perylene-3-ylethynyl)uracil-1)acetyl] amino}triazol-1-yl)-2-oxapent-1-yl]} methane (**9e**)

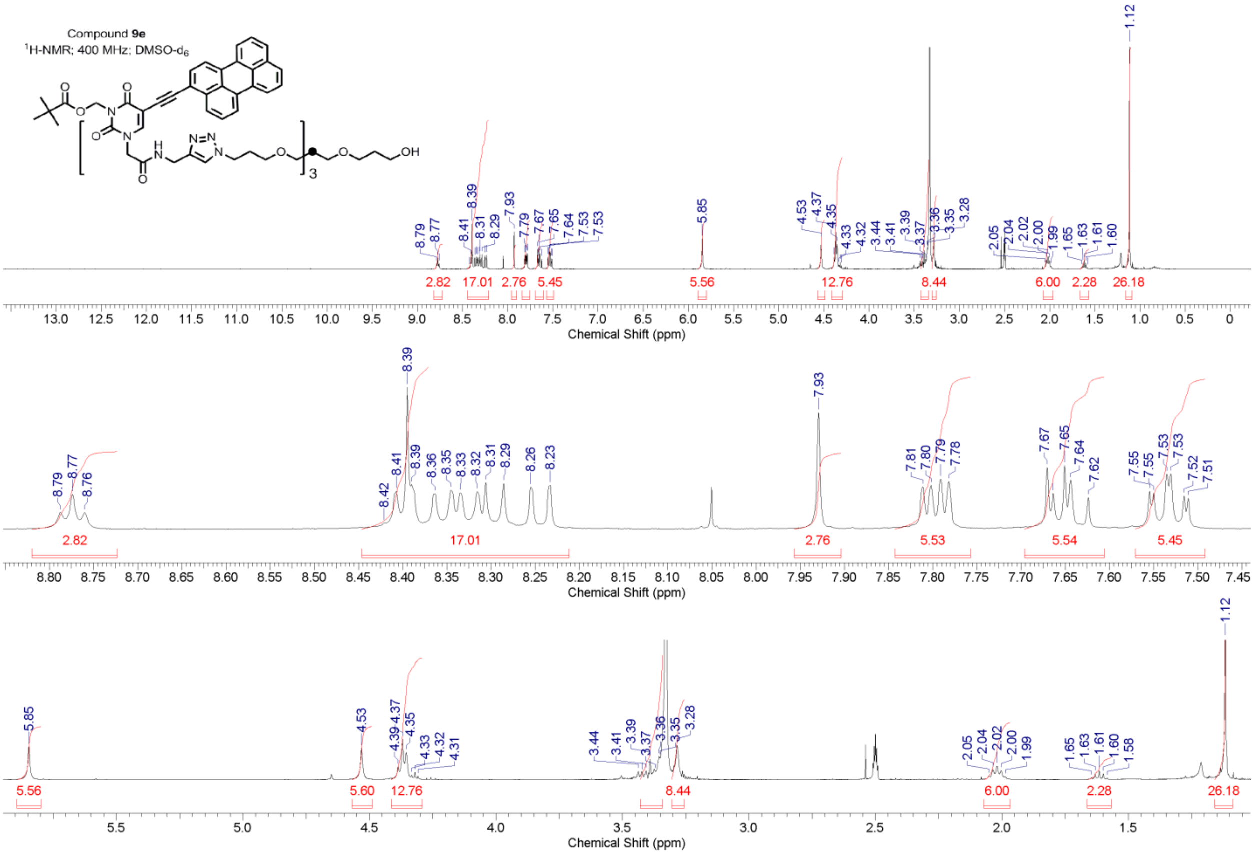

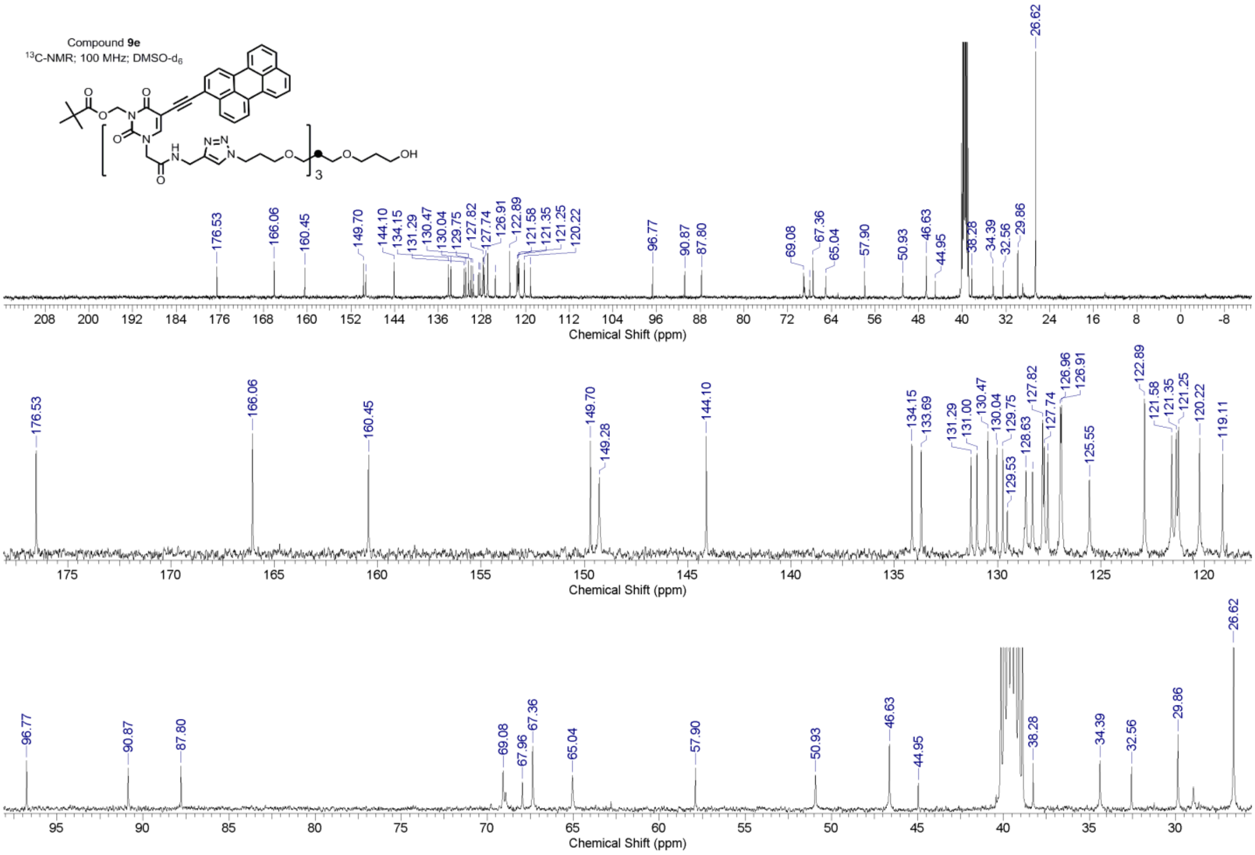

NMR spectra of tetrakis [5-(4-{N-[(3-(Pivaloyloxymethyl)-5-(perylen-3-ylethynyl) uracil-1)acetyl]amino}triazol-1-yl)-2-oxapent-1-yl] methane (**9f**)

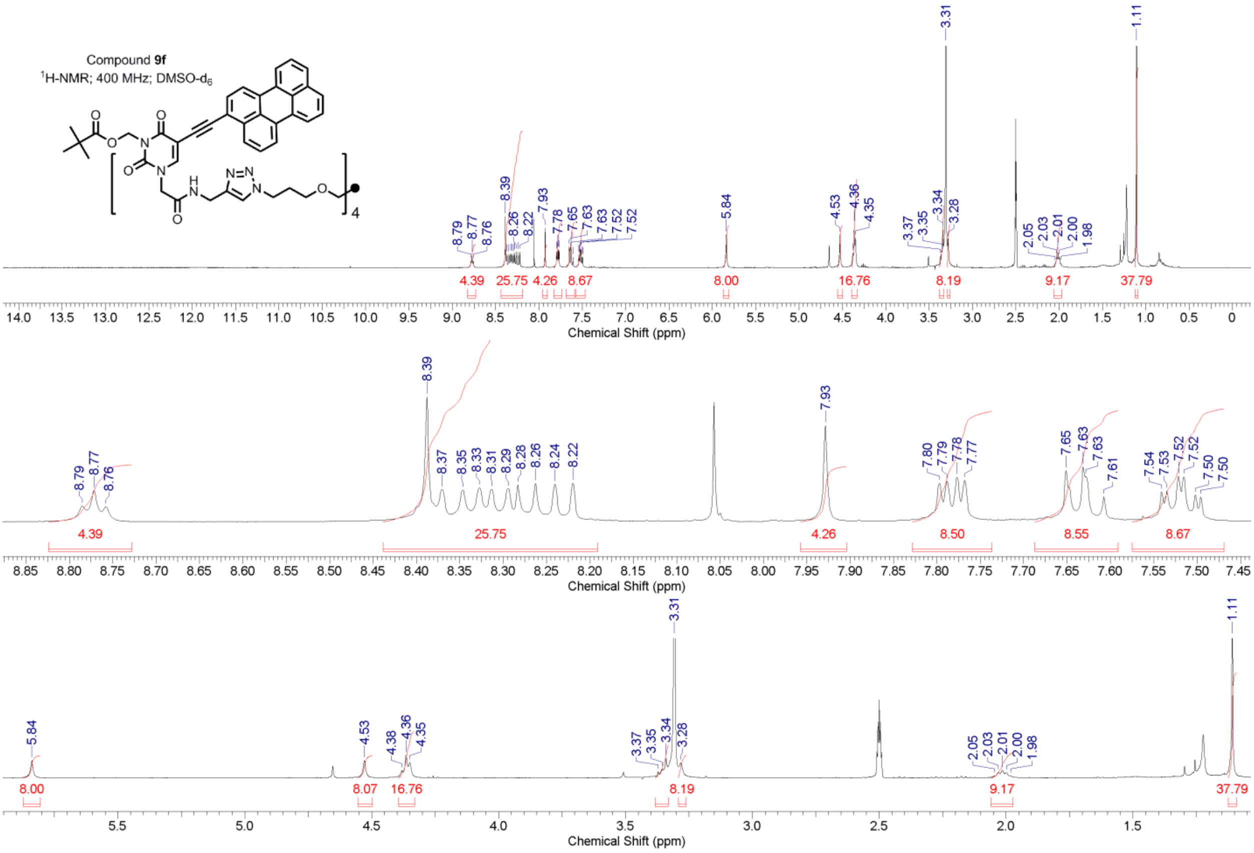

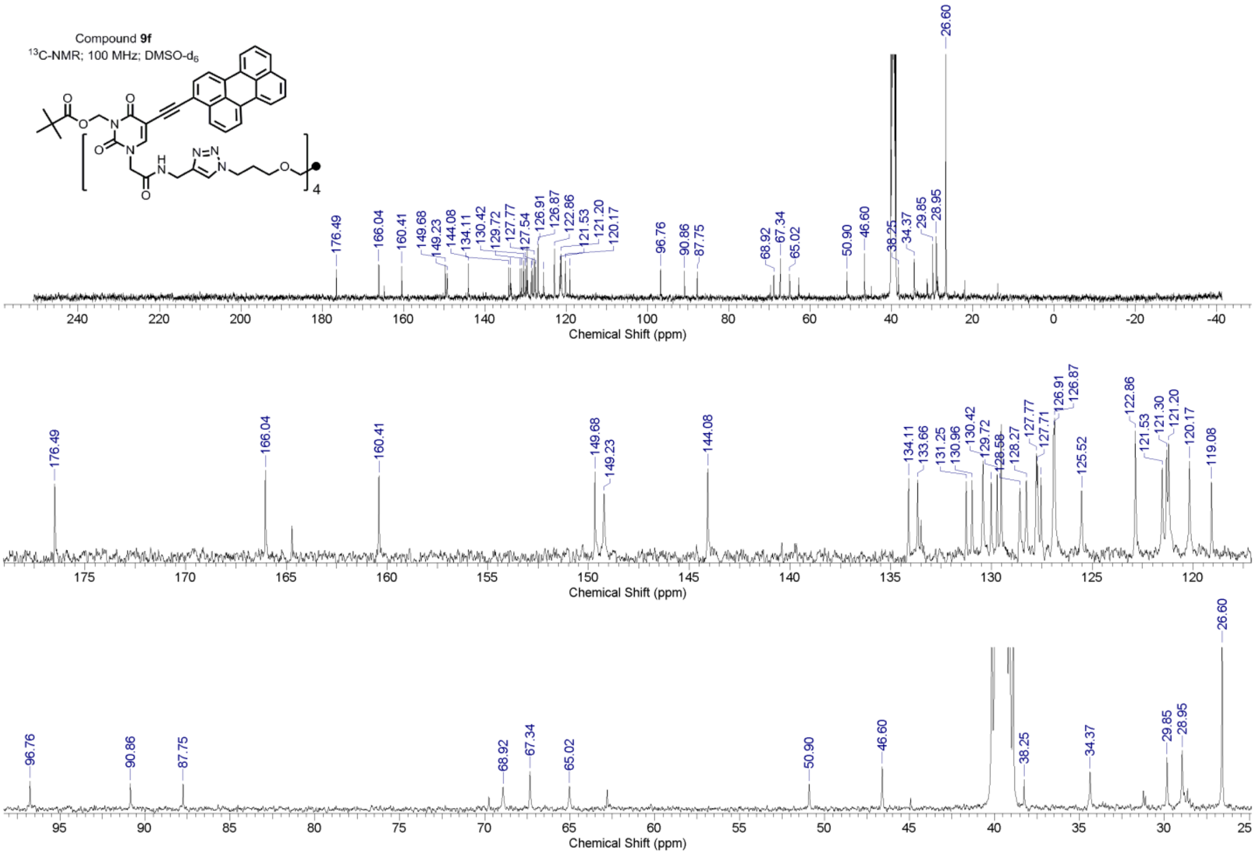

NMR spectra of (5-(perylen-3-ylethynyl)uracil-1)-N-propargyl acetamide (**8**)

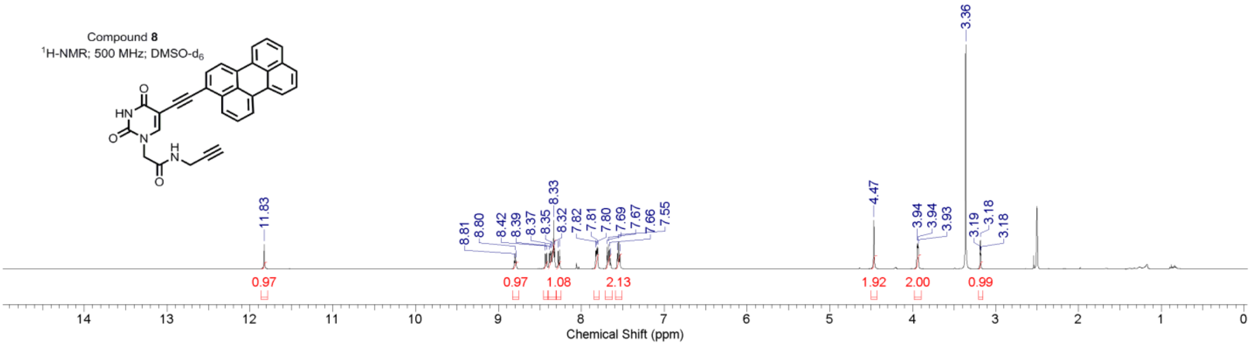

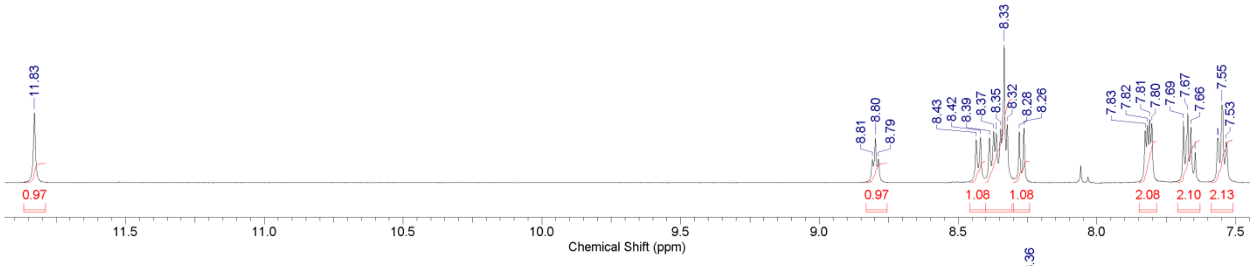

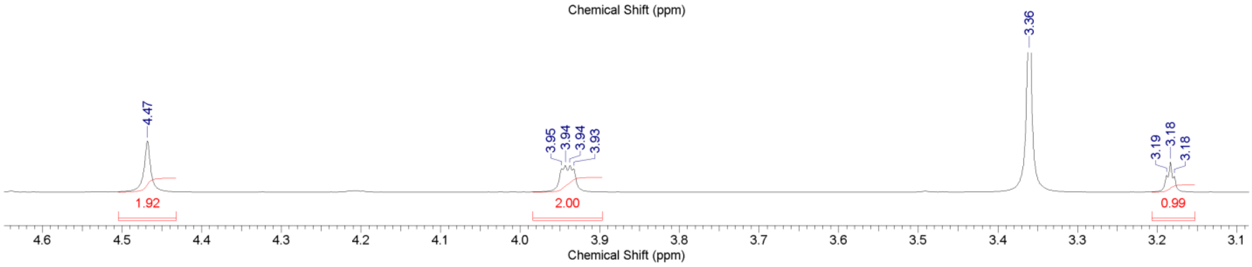

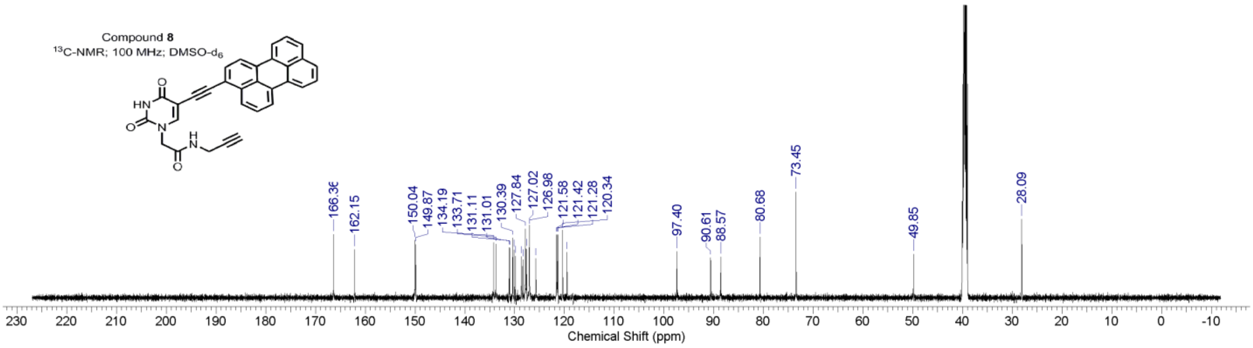

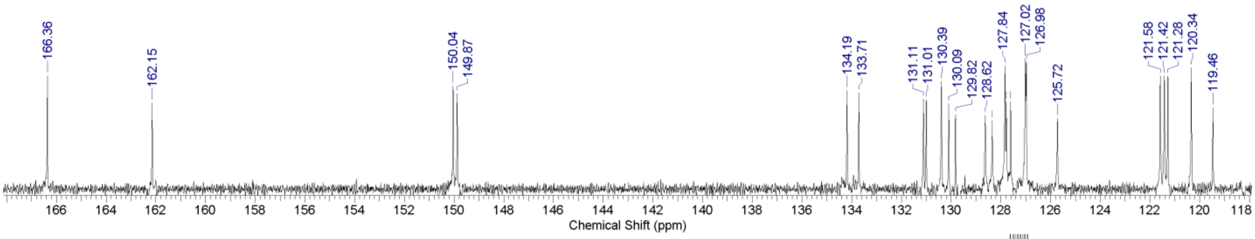

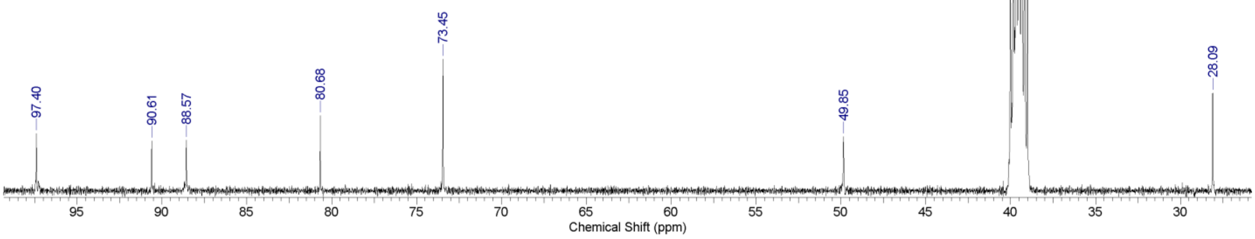

NMR spectra of 5-(perylen-3-ylethynyl)uracil-1)-N-(1-benzyltriazol-4-yl) acetamide (**10a**)

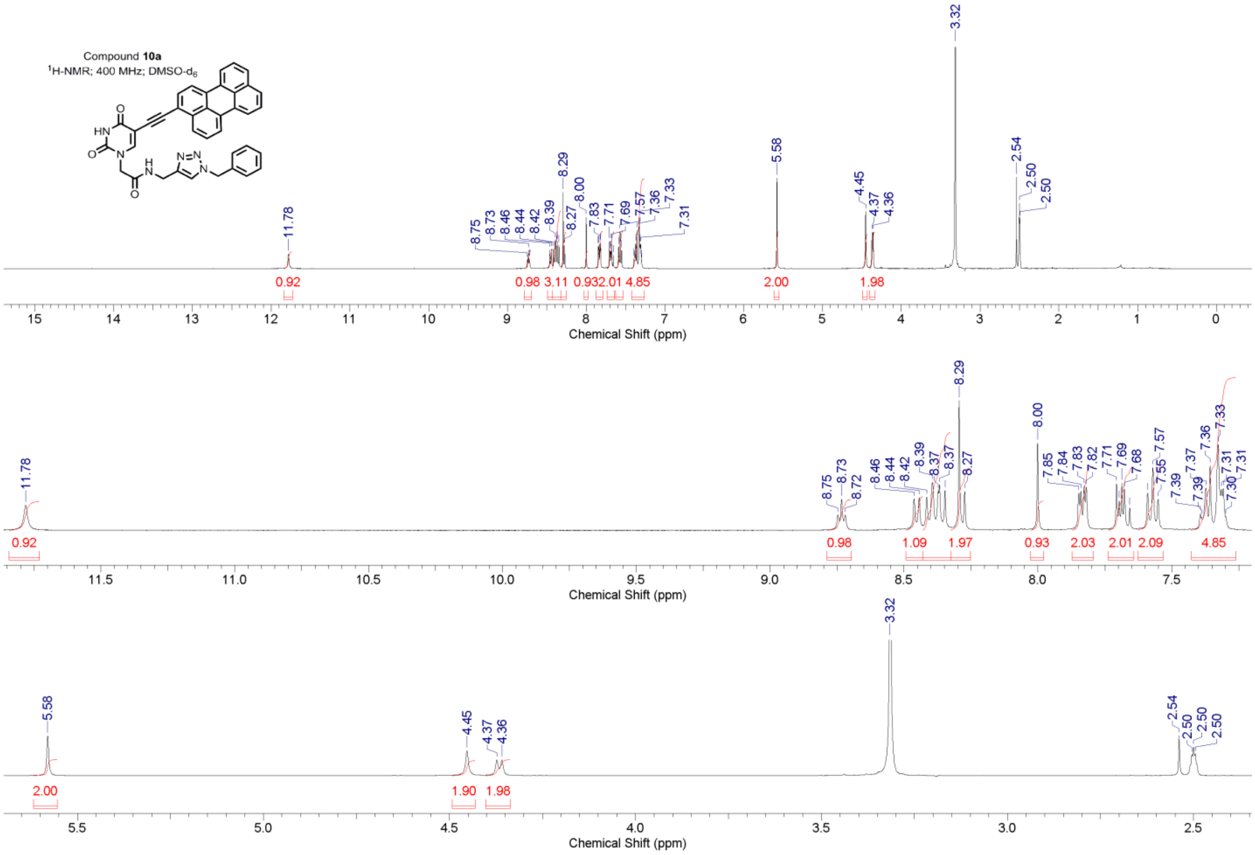

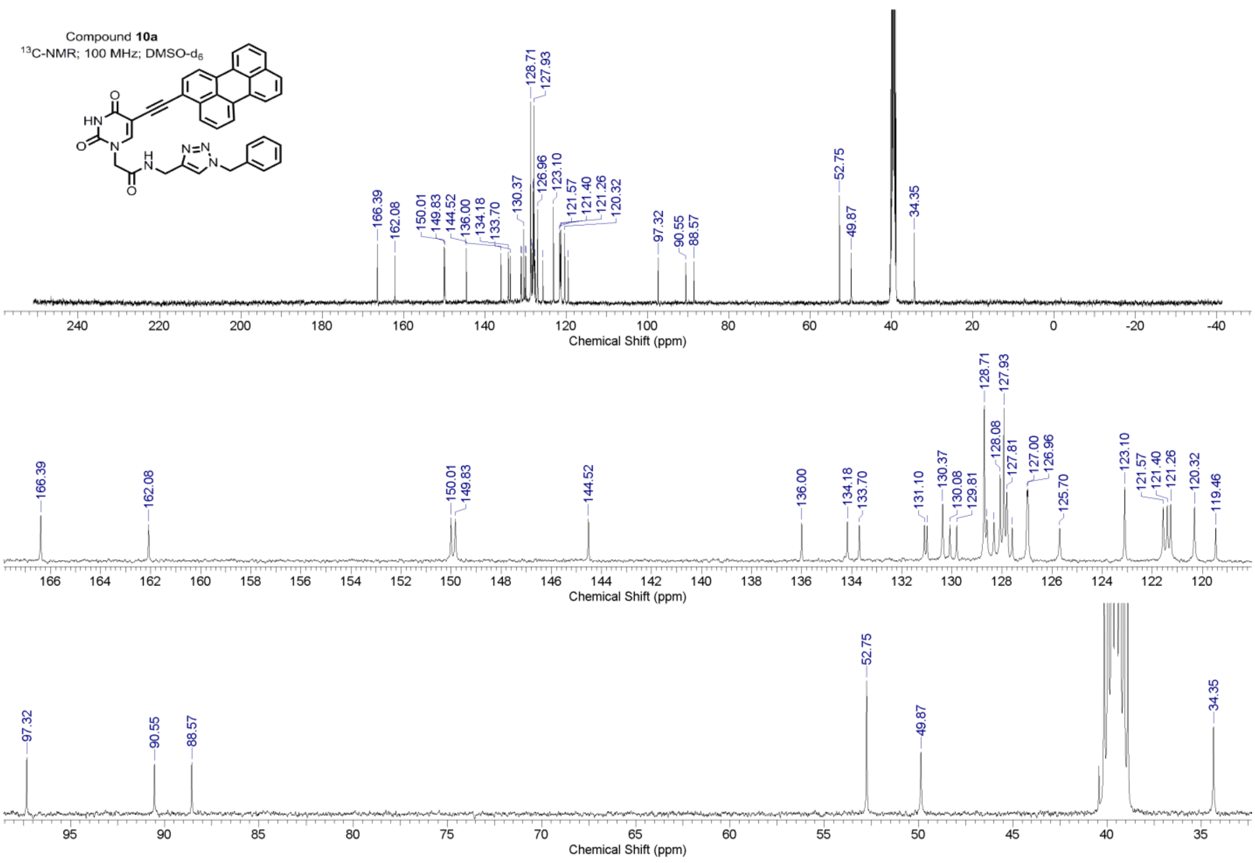

NMR spectra of 5-(perylen-3-ylethynyl) uracil-1)-N-(1-hydroxyethyltriazol-4-yl) acetamide (**10b**)

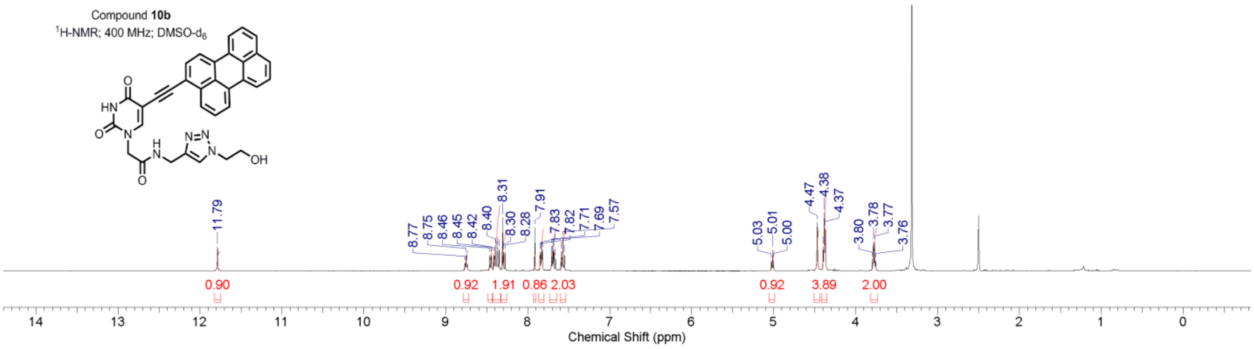

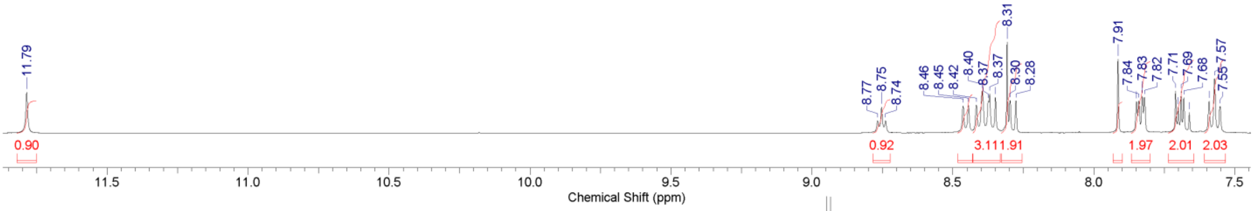

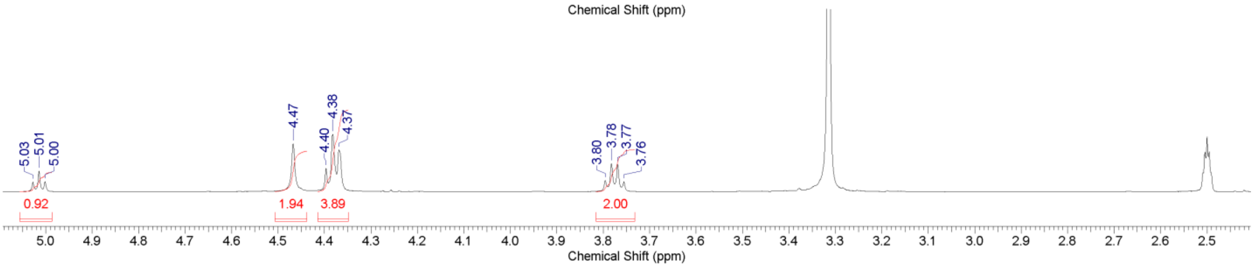

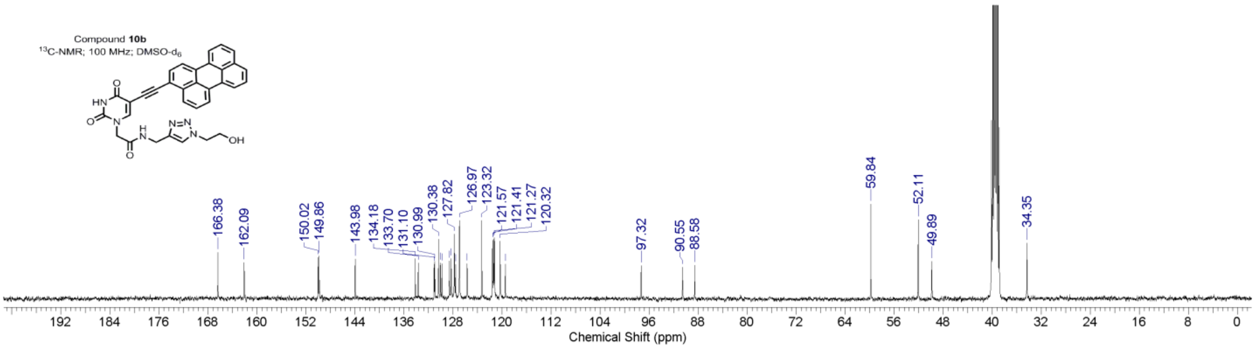

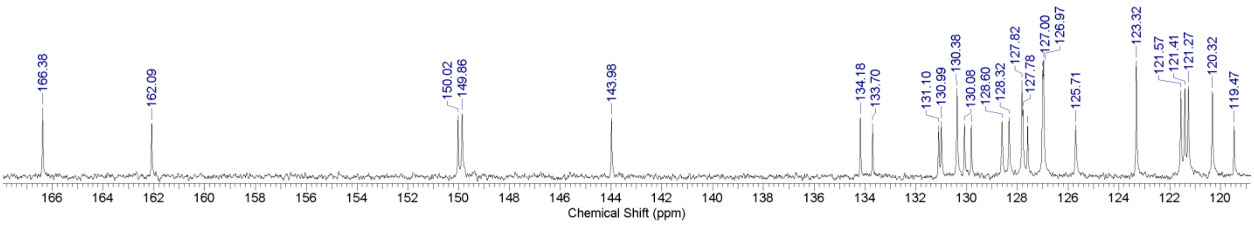

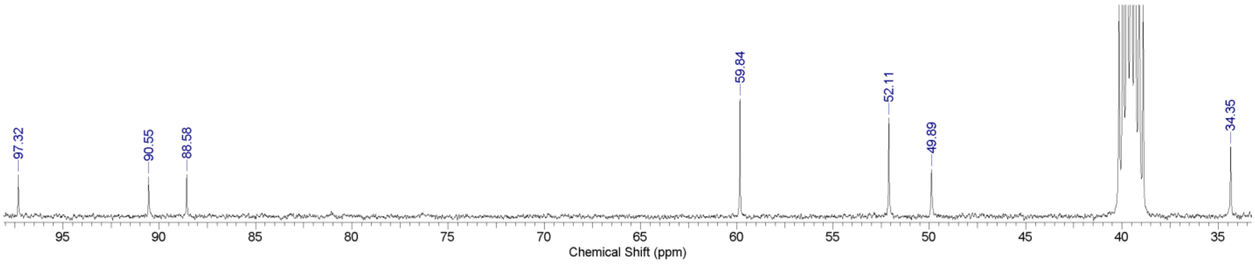

NMR spectra of 5-(perylen-3-ylethynyl) uracil-1)-N-(1-{1,1,1-[tris(5-hydroxy-2-oxapentyl)]-3-oxahex-6-yl} triazol-4-yl) acetamide (**10c**)

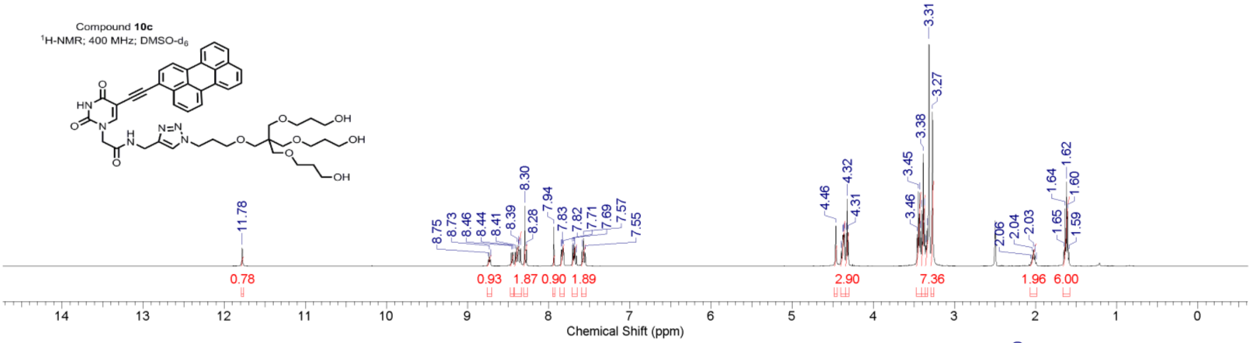

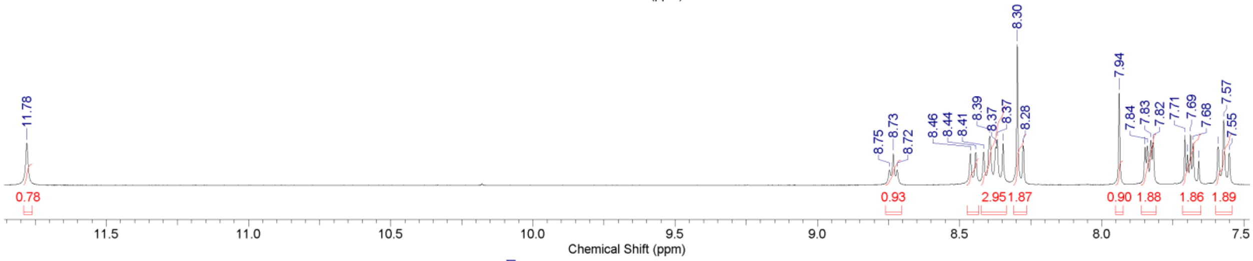

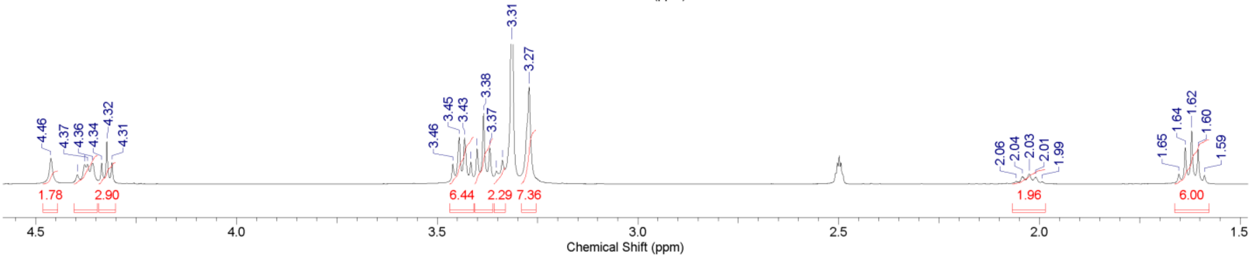

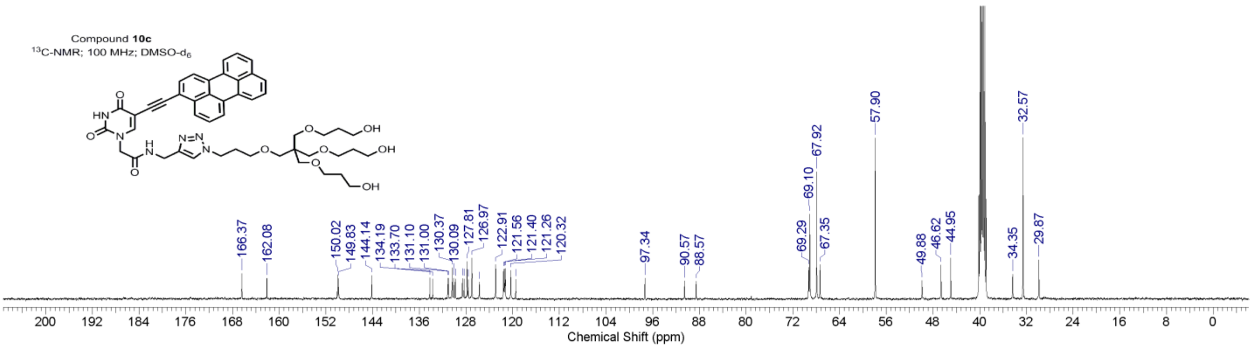

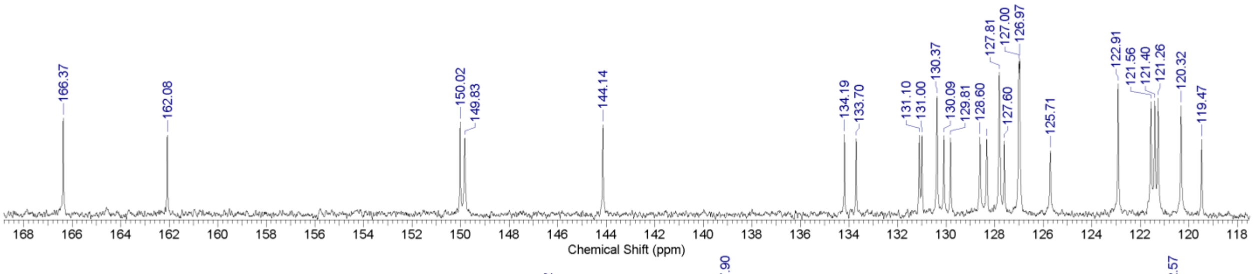

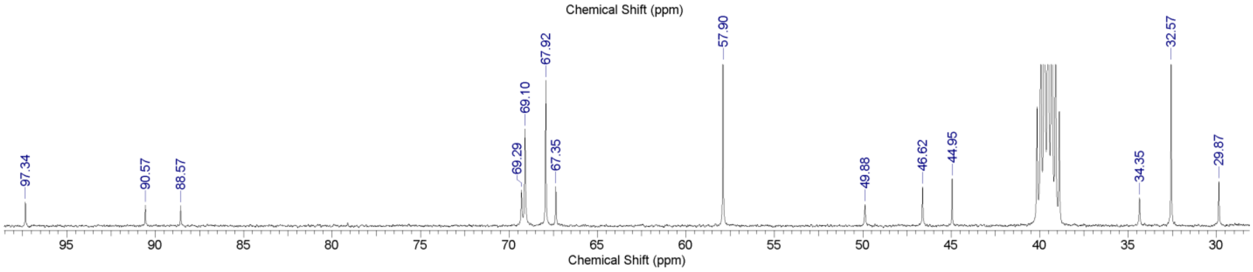

High resolution mass spectra of synthesized compounds

(3-(Pivaloyloxymethyl)-5-(perylen-3-ylethynyl)uracil-1)-N-propargyl acetamide (**7**)

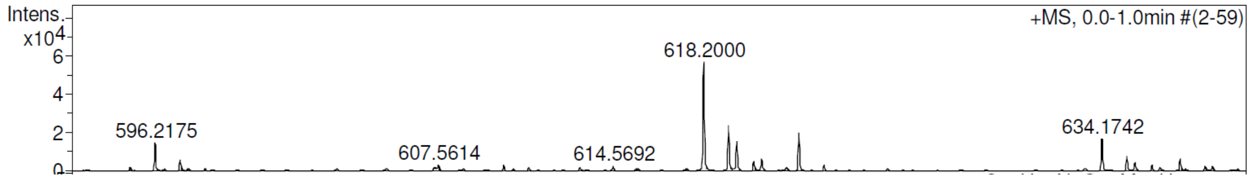

5-(Perylen-3-ylethynyl)uracil-1)-N-propargyl acetamide (**8**)

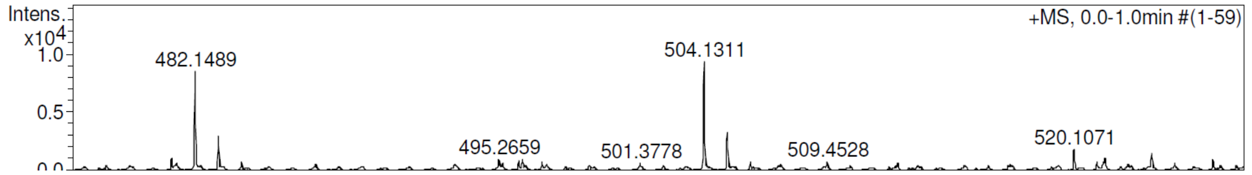

(3-(Pivaloyloxymethyl)-5-(perylen-3-ylethynyl)uracil-1)-N-(1-benzyltriazol-4-yl)acetamide (**9a**)

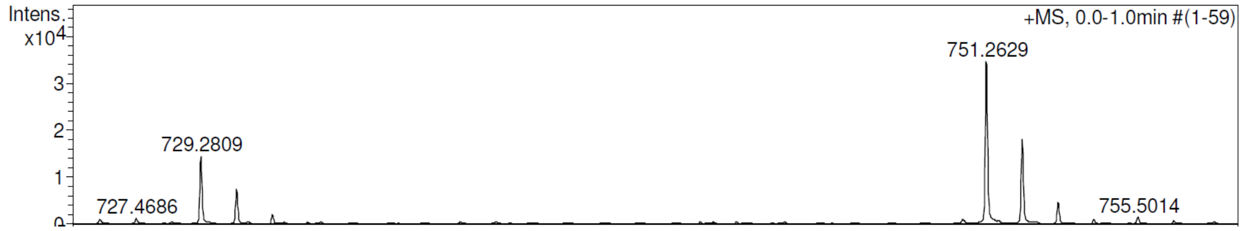

3-(Pivaloyloxymethyl)-5-(perylen-3-ylethynyl)uracil-1)-N-(1-hydroxyethyltriazol-4-yl)acetamide (**9b**)

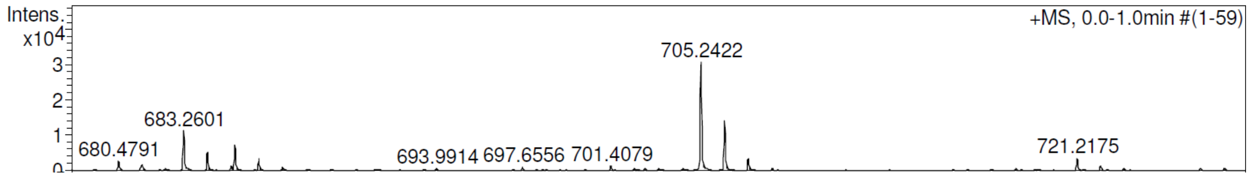

(3-(Pivaloyloxymethyl)-5-(perylen-3-ylethynyl)uracil-1)-N-(1-{1,1,1-[tris(5-hydroxy-2-oxapentyl)]-3-oxahex-6-yl}triazol-4-yl)acetamide (**9c**)

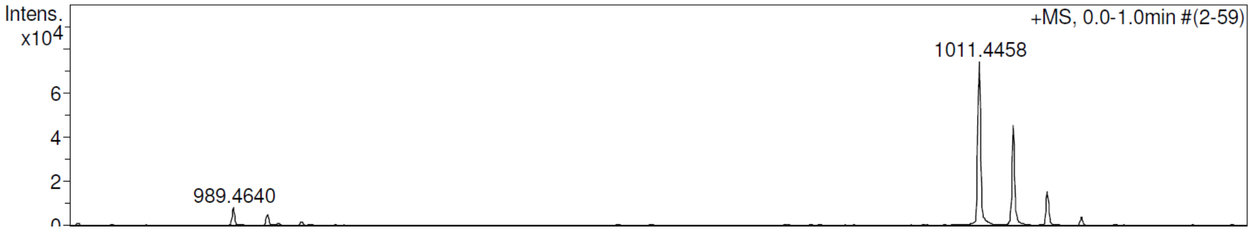

1,1-[bis(5-hydroxy-2-oxapentyl)]-1,1-{bis[5-(4-{N-[(3-(pivaloyloxymethyl)-5-(perylen-3-ylethynyl)uracil-1)acetyl]amino}triazol-1-yl)-2-oxapent-1-yl]} methane (**9d**)

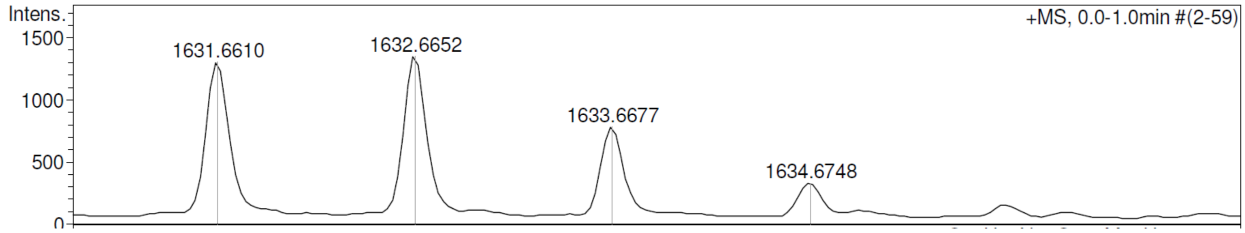

1-(5-hydroxy-2-oxapentyl)-1,1,1-{tris[5-(4-{N-[(3-(pivaloyloxymethyl)-5-(perylen-3-ylethynyl)uracil-1)acetyl] amino}triazol-1-yl)-2-oxapent-1-yl]} methane methane (**9e**)

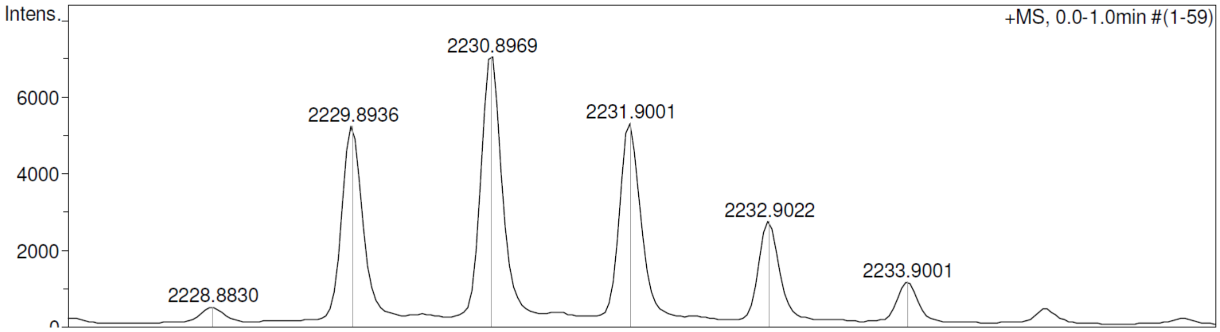

tetrakis [5-(4-{N-[(3-(pivaloyloxymethyl)-5-(perylen-3-ylethynyl) uracil-1)acetyl]amino}triazol-1-yl)-2-oxapent-1-yl] methane (**9f**)

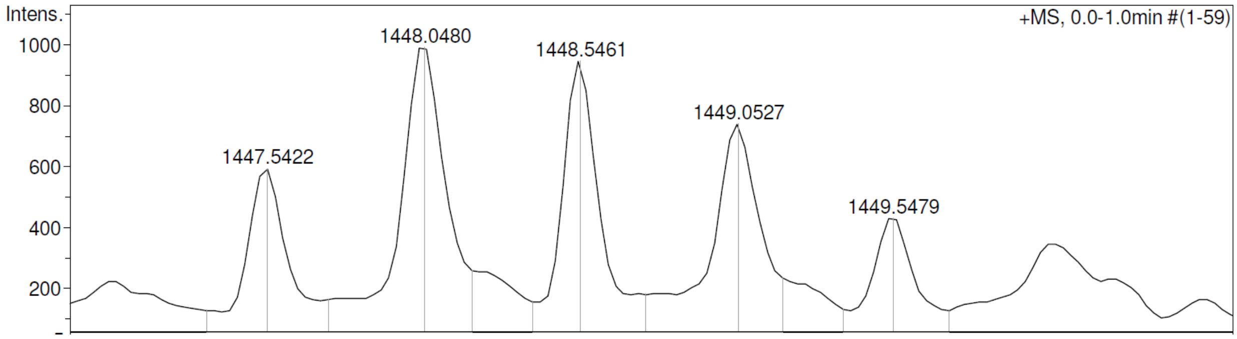

5-(perylen-3-ylethynyl)uracil-1)-N-(1-benzyltriazol-4-yl) acetamide (**10a**)

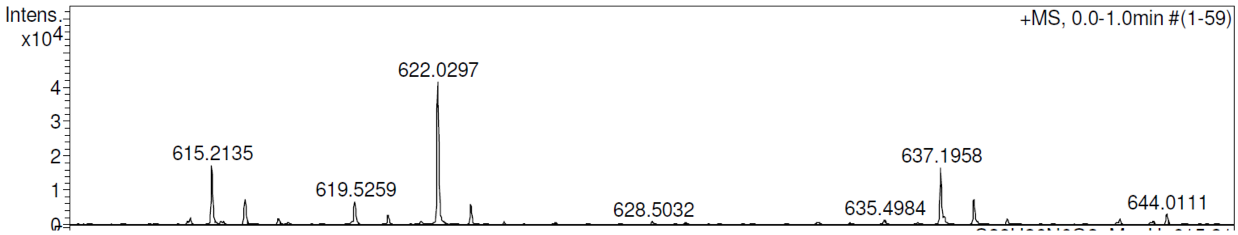

5-(perylen-3-ylethynyl) uracil-1)-N-(1-hydroxyethyltriazol-4-yl) acetamide (**10b**)

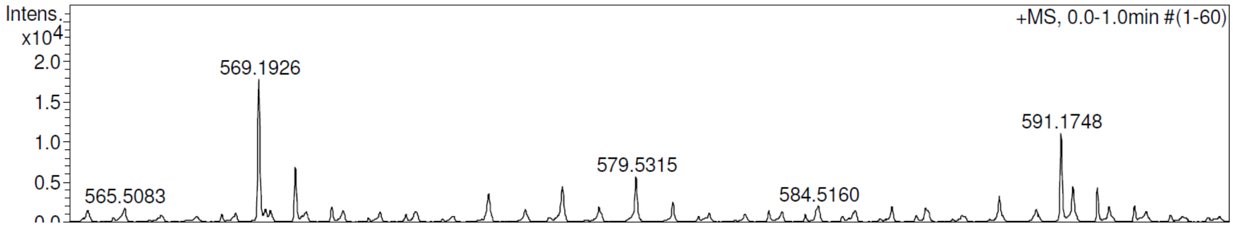

5-(perylen-3-ylethynyl) uracil-1)-N-(1-{1,1,1-[tris(5-hydroxy-2-oxapentyl)]-3-oxahex-6-yl} triazol-4-yl) acetamide (**10c**)

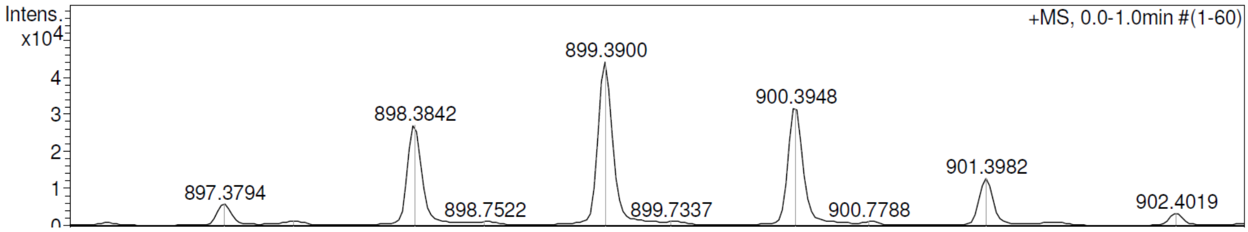

Supplement: RA-009-C9RA06313G-s001 [file RA-009-C9RA06313G-s001.pdf]
